# Oxetanes in Drug Discovery Campaigns

**DOI:** 10.1021/acs.jmedchem.3c01101

**Published:** 2023-09-07

**Authors:** Juan J. Rojas, James A. Bull

**Affiliations:** Department of Chemistry, Imperial College London, Molecular Sciences Research Hub, White City Campus, Wood Lane, London W12 0BZ, U.K.

## Abstract

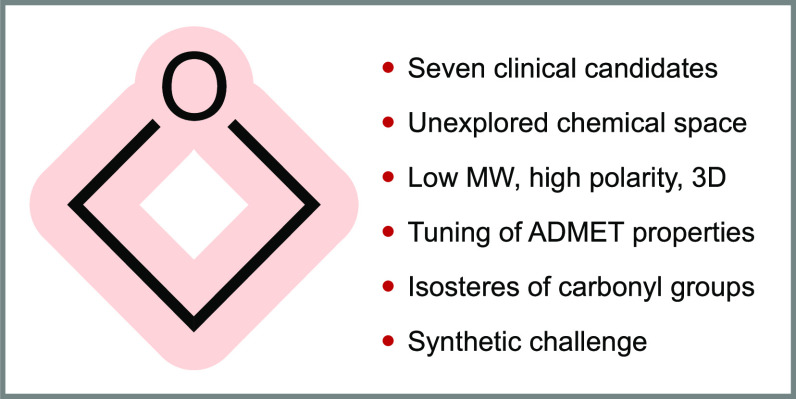

The oxetane ring is an emergent, underexplored motif in drug discovery that shows attractive properties such as low molecular weight, high polarity, and marked three-dimensionality. Oxetanes have garnered further interest as isosteres of carbonyl groups and as molecular tools to fine-tune physicochemical properties of drug compounds such as p*K*_a_, LogD, aqueous solubility, and metabolic clearance. This perspective highlights recent applications of oxetane motifs in drug discovery campaigns (2017–2022), with emphasis on the effect of the oxetane on medicinally relevant properties and on the building blocks used to incorporate the oxetane ring. Based on this analysis, we provide an overview of the potential benefits of appending an oxetane to a drug compound, as well as potential pitfalls, challenges, and future directions.

## Significance

Oxetanes have gained significant interest in medicinal chemistry as small, polar, and 3-dimensional motifs with potential as isosteres of carbonyl groups. This perspective analyzes recent applications of oxetanes in drug discovery, covering the benefits of appending the oxetane motif, synthetic strategies employed, and potential pitfalls, challenges, and future directions, to serve as a guide for medicinal chemists considering the inclusion of oxetane rings in current and future drug discovery campaigns.

## 1 Introduction

As programs in medicinal chemistry seek to focus on ever more challenging biological targets, the molecular complexity of drug candidates has increased substantially in the last 50 years.^1^ Although the quantification of “complexity” is debated,^1,2^ a general consensus is that more complex molecular structures display more three-dimensionality (i.e., not flat) and contain a higher degree of sp^3^-hybridized carbon atoms. There is a significantly lower attrition rate of “nonflat” clinical candidates,^3^ which has been attributed to higher target selectivity^4^ and superior pharmacokinetic (PK) and toxicity profiles.^5^ Consequently, practitioners in drug discovery are also in an ongoing search for new but validated molecular motifs that can beneficially modulate the binding and physicochemical properties of a compound and offer intellectual property (IP) advantages.

**Figure 1:**
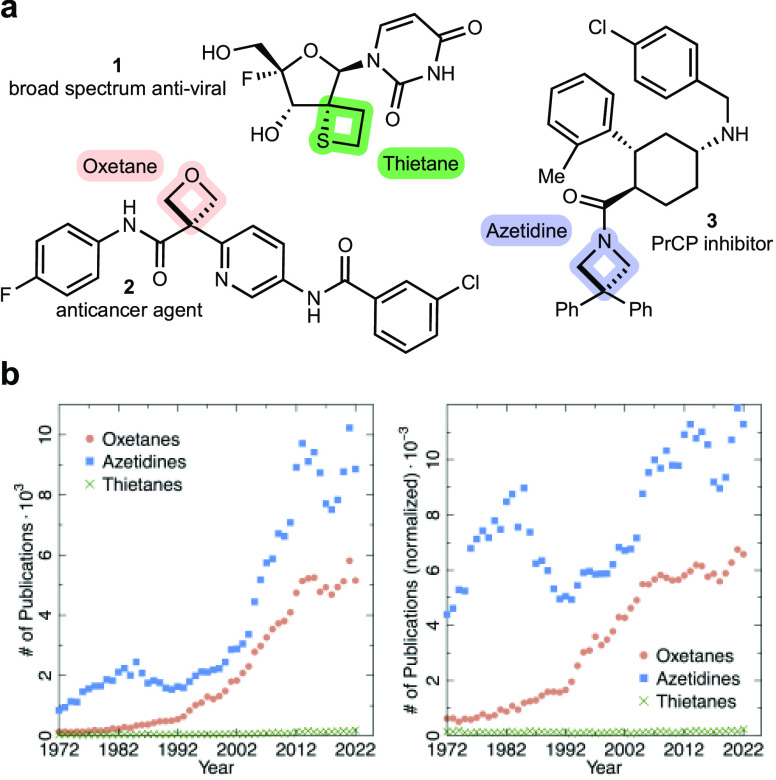
(a) Examples of bioactive four-membered heterocycles.^8^ (b) Appearance of four-membered heterocycles in the literature.^9^ (Left) Absolute number of publications. (Right) Normalized against the total number of publications recorded per year. Thietanes also include the sulfoxide and sulfone oxidation states. PrCP = prolylcarboxypeptidase.

Four-membered heterocycles have emerged as beneficial motifs because of their low molecular weight, high polarity, and three-dimensionality, which can improve properties including target affinity and aqueous solubility (**1**–**3**, Figure 1a).^6,7^ These features have led to an increase in popularity of heterocyclic four-membered rings in the last 30 years, as is observed in the relative surge in publications since 1992 (Figure 1b, right; also see the Supporting Information for thietanes alone).

**Figure 2:**
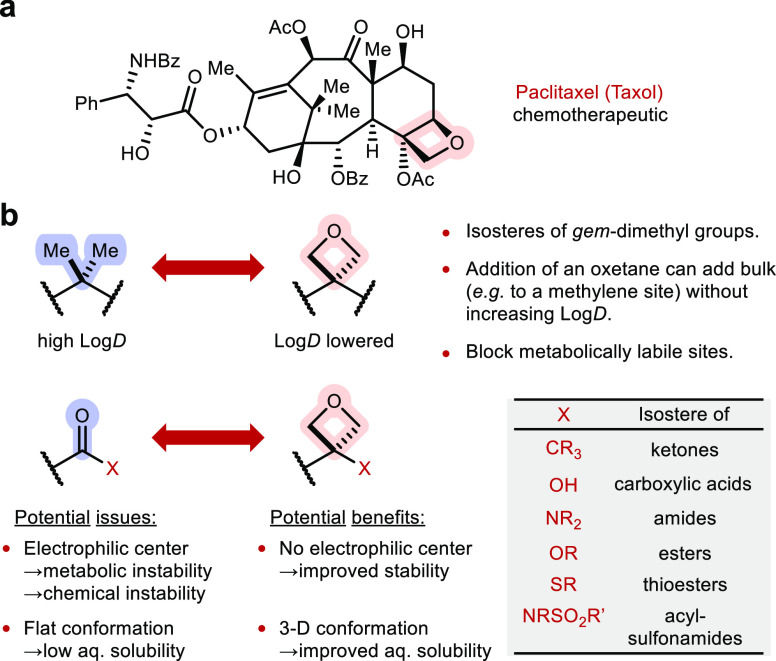
(a) Taxol. (b) Oxetanes as potential isosteres of *gem*-dimethyl groups and carbonyl derivatives.

The oxetane scaffold has gained particular interest among the synthetic and medicinal chemistry communities.^10^ Oxetanes also have a relatively high occurrence in natural products compared to other four-membered heterocycles.^11^ The best-known oxetane natural product is paclitaxel, which is also the only FDA-approved bioactive oxetane compound (Figure 2a).^12,13^ Commonly known by its brand name, Taxol, paclitaxel used to be the best-selling anticancer drug and is still the front line agent for the treatment of breast cancer and is part of the WHO List of Essential Medicines.^14^ Although the oxetane ring was shown not to be strictly essential for the bioactivity of Taxol, nonoxetane analogues displayed lower binding affinity and cytotoxicity than the parent Taxol structure.^13b,13c^ Oxetanes with a 3,3-disubstitution pattern have been validated as surrogates or isosteres of *gem*-dimethyl and carbonyl functionality and are becoming used as an example of modern isosterism in medicinal chemistry (Figure 2b).^15^ In a series of influential reports, the Carreira group in collaboration with Hoffmann-La Roche (Roger-Evans, Müller) demonstrated that oxetanes can be used instead of *gem*-dimethyl groups to block C–H metabolic weak spots in a drug candidate, without the unfavorable increase in lipophilicity associated with the latter.^16^ Concerning carbonyls, oxetane analogues of ketones have shown potential to improve metabolic stability (substrate-dependent) and increase three-dimensionality, while maintaining comparable H-bonding ability, dipole moment, and lone pair orientation,^16b,16c,17^ Amino-oxetanes have found notable applications as peptidomimetics, with the oxetanyl structure showing improved stability against enzymatic degradation while maintaining bioactivity.^18^ Other oxetane derivatives such as oxetanols,^19^ oxetane sulfides,^20^ oxetane ethers,^21^ and oxetane sulfonamides^22^ have also been proposed as isosteres and evaluated to some extent versus carboxylic acids, thioesters, esters, and *N*-acylsulfonamides, respectively (Figure 2b).

In addition to the attractive properties applicable to all four-membered heterocycles (low molecular weight, polarity, increased three-dimensionality), the electronegative oxygen atom confers oxetanes with a powerful inductive electron-withdrawing effect that propagates to the 3-position through two short σ-bonding frameworks. As such, it was demonstrated that an oxetane α to an amine reduces the p*K*_aH_ of the amine by 2.7 units (that is, ca. 500 times less basic) from 9.9 to 7.2 by means of its negative inductive effect.^16c^ Additionally, it was recently shown by Hayes and co-workers (AstraZeneca) that selected oxetane compounds were degraded by the human microsomal epoxide hydrolase (mEH).^23^ This was the first example of a nonepoxide substrate being metabolized by mEH, and it could have potential applications to avoid clearance by cytochrome P450 enzymes (CYPs), which can be problematic due to undesired and poorly predictable drug–drug interactions that can cause liver toxicity on comedication.^23,36b^

The ring-strain associated with small rings coupled with the electronegative oxygen atom also render oxetane substrates potentially unstable to ring-opening degradative processes, particularly under acidic conditions. The anecdotal categorical instability of oxetanes to acidic conditions is, however, a misconception. Oxetane stability is often dictated by its substitution pattern, whereby 3,3-disubstituted examples are most stable because the path of external nucleophiles to the C–O σ* antibonding orbital is sterically blocked by the substituents (Figure 2b).^16c^ Observations of specific instability persist, which can also be dependent upon local structural features,^24^ including the presence or absence of other basic sites. Oxetanes substituted with electron-donating groups at C2 are likely to be unstable. Internal nucleophiles can also lead to cyclization processes, which can be synthetically productive.^24,25^

The “rediscovery” of the oxetane ring from 2006 fueled its inclusion into drug discovery programs. However, despite the potential benefits on molecular properties in using an oxetane ring in drug design, a dearth of synthetic methods continued to limit applications in drug-like compounds. This challenge has encouraged symbiotic efforts in academia and industry to enable their efficient inclusion into target compounds. Notable advances have been reported on the synthesis of oxetanes^10,26,27^ and in the functionalization of the intact ring.^28−30^ Together, these have further facilitated the investigation of oxetanes in drug discovery programs, which are beginning to bear fruit.

Applications as part of such campaigns, including the patent literature, have been comprehensively covered in reviews up to 2016 with case studies.^6,10^ Here, we present a perspective on recent developments (2017–2022), focusing on the effects of substituting an oxetane ring into a drug compound. This includes discussion on seven oxetane-containing compounds that are currently in clinical trials (as of January 2023), highlighting potential successes. We also analyze the most popular sources of oxetane used for functionalization and the implications on structural patterns in the drug compounds. We discuss the potential benefits, pitfalls, and challenges of including an oxetane motif in drug design.

## 2 Applications of Oxetane MoTIFS

### 2.1 Literature Search

**Figure 3:**
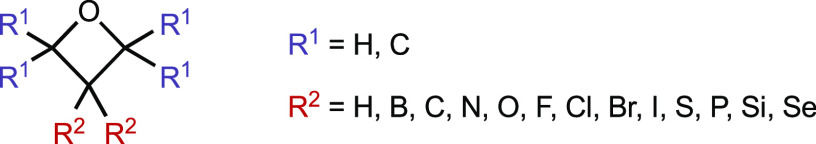
Substructure used for literature search.

Drug candidates that contain an oxetane motif currently undergoing clinical trials (stages I–III) were identified using the Drug Bank platform, excluding β-lactones and taxane derivatives.^31^ A Scifinder search was then conducted (January 2023) with an oxetane ring as a substructure, excluding heteroatomic substitution in the 2-position (i.e., also excluding β-lactones; Figure 3). Results were then filtered to include only: biological study, therapeutic use, pharmacological activity, biological study (unclassified), pharmacokinetics, and biological use (unclassified), published in English between 2017–2022. The search recorded 6007 patents that included an oxetane compound with reported biological activity.

For journal articles, results were limited to the following medicinal chemistry journals: *ACS Medicinal Chemistry Letters*, *Bioorganic and Medicinal Chemistry*, *Bioorganic and Medicinal Chemistry Letters*, *ChemMedChem*, *The European Journal of Medicinal Chemistry*, *The Journal of Medicinal Chemistry*, *Medicinal Chemistry Research*, *MedChemComm*, and *RSC Medicinal Chemistry*. The results (449 articles) were then manually triaged to those that included a synthetic oxetane compound (e.g., not from the taxane family) in the optimization campaign (198 articles). The effect of oxetane introduction as well as the source of oxetane were analyzed (where this information was available). See the Supporting Information for the full list of references.

### 2.2 Clinical Candidates

**Figure 4:**
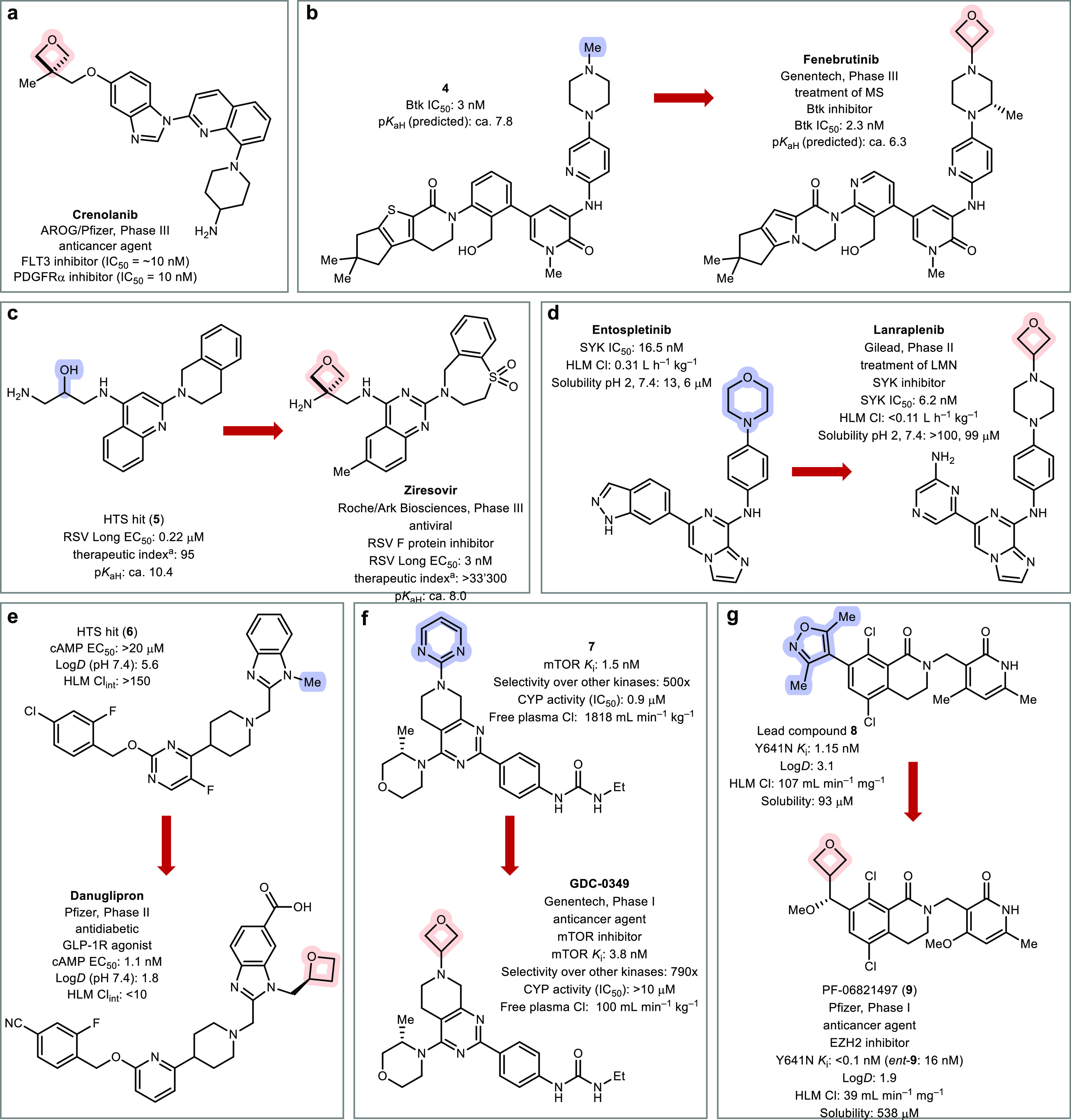
Fully synthetic (nontaxane-related) drug candidates containing an oxetane scaffold and effect of introducing the oxetane ring (where information available).^32−38^ (a) Crenolanib (no information on the discovery campaign),^32^ (b) fenebrutinib,^33^ (c) ziresovir,^34^ (d) lanraplenib,^35^ (e) danuglipron,^36^ (f) GDC-0349,^37^ and (g) PF-06821497 (**9**).^38a^ Btk = Bruton’s tyrosine kinase; cAMP = cyclic adenosine monophosphate; Cl = clearance; Cl_int_ = intrinsic clearance; FLT3, fms like tyrosine kinase 3; HLM = human liver microsomes; HTS = high-throughput screening; PDGFRα, platelet-derived growth factor receptor α; and Y641N, mutant form of EZH2. ^a^ Therapeutic index (TI) = CC_50_/EC_50_. CC_50_ = concentration of compound that manifests cytotoxicity toward 50% of the uninfected HEp-2 cells.^34^

There are currently seven oxetane-containing drug candidates undergoing clinical trials.^31^ In phase III are crenolanib (Figure 4a), developed by AROG Pharmaceuticals/Pfizer for treatment of various types of cancer [acute myeloid leukemia (AML), gastrointestinal stromal tumor (GIST), glioma];^32^ fenebrutinib (Figure 4b), developed by Genentech as a treatment for multiple sclerosis (MS);^33^ and ziresovir (Figure 4c), developed by Hoffmann–La Roche/Ark Biosciences for treatment of respiratory syncytial virus (RSV).^34^ Lanraplenib [Figure 4d, Gilead Sciences, treatment of Lupus Membranous Nephropathy (LMN)]^35^ and danuglipron (Figure 4e, Pfizer, treatment of diabetes)^36^ are in phase II, and GDC-0349 (Figure 4f, Genentech, treatment of Non-Hodgkin’s lymphoma and solid tumors)^37^ and PF-06821497 [Figure 4g, Pfizer, treatment of relapsed/refractory SCLC (small cell lung cancer), prostate cancer, and follicular lymphoma]^38^ in phase I. It is notable that over 50% of the structures are amino-oxetanes (4/7), whereby the oxetane motif will attenuate amine basicity. Additionally, in six out of seven compounds the oxetane is substituted in the 3-position, likely due to superior stability and/or higher synthetic tractability. Generally, the oxetane ring was introduced during the late stages of the drug discovery campaigns to improve unsatisfactory PK properties (most often LogD, solubility, clearance, or basicity) of the lead compounds (Figure 4b–g; no information available on crenolanib).

In fenebrutinib, the oxetane motif was an essential component of the drug, introduced during midstages of the discovery campaign to lower the p*K*_aH_ of the piperazine ring from 7.8 (**4**) to 6.3 (Figure 4b).^39^ Compound series such as **4** and analogues suffered from high hepatotoxicity in rat and dog pilot studies.^33a^ This toxicity issue was overcome by replacing the core phenyl ring in **4** by a pyridine motif (fenebrutinib), which lowered LogD by >1 unit. The change in heterocycle from thiophene to pyrrole provided a better fit into the H3 selectivity pocket of Btk and increased potency. During the late stages of the campaign, significant efforts were spent to replace the oxetane ring, but all nonoxetane analogues showed inferior solubility and pharmacokinetic properties.^33a^ Instead, the addition of a methyl group to the piperazine ring increased van der Waals contacts with the protein and skewed the piperazine out of the plane of the adjacent arene, inducing a ca. 2-fold improvement in potency.

The oxetane moiety in ziresovir, deemed the “highlight of the discovery”,^34b^ was introduced at a late stage in the discovery campaign, to reduce the basicity of the terminal amine in amino-alcohol **5**, which was important to lower the volume of distribution (*V*_ss_) to avoid its undesired accumulation in tissue and minimize risks of toxicity (Figure 4c).^34^ Basic functional groups were speculated to interact strongly with acidic phosphatidylserine in lung tissue. A docking model suggested the oxetane ring not be involved in any interactions with protein residues.^34a^ Instead, the oxetane served as a conformational and basicity control. Lower potency and therapeutic indexes (TI = CC_50_/EC_50_) were observed with other linkers such as *gem*-dimethyl (EC_50_ = 16 nM; TI = 1,250), cyclopropyl (EC_50_ = 4 nM; TI = 3,250), and cyclobutyl (EC_50_ = 100 nM; TI = 210). Expanding the six-membered tetrahydroisoquinoline ring in alcohol **5** to the seven-membered ring in Ziresovir increased the dihedral angle between the two aromatic systems from ca. 40 to 90°, increasing overall three-dimensionality and potency. Introduction of the sulfone moiety in ziresovir blocked an important metabolic soft spot and reduced clearance.

Entospletinib is a potent SYK (spleen tyrosine kinase) inhibitor that was recently withdrawn from clinical development due to insufficient solubility, adverse drug–drug interactions with proton pump inhibitors, and high metabolic clearance by oxidation of the morpholine ring (Figure 4d).^35a^ A late-stage drug optimization campaign was thus conducted to improve the unsatisfactory ADME properties of entospletinib. Exchanging morpholine for 4-ethyl-piperazine improved metabolic stability, but the increased basicity (calcd p*K*_aH_ = 8.0) led to poor selectivity of T- versus B-cells (T/B ratio = 5). Introduction of an oxetane on the 4-position instead of the ethyl group doubled selectivity (T/B ratio = 10) by reducing basicity (calcd p*K*_aH_ = 6.4), while keeping the increased metabolic stability and also showing high solubility at pH 2 and Caco-2 permeability. A cocrystal structure of lanraplenib and the SYK kinase domain (PDB code 6VOV) revealed the *N*-oxetane-piperazine part to occupy a solvent-accessible space outside the protein pocket. In the final optimization, the indazole ring in entospletinib was exchanged for an amino-pyrazine to reduce aromatic count and increase three-dimensionality. This change reduced LogD from 2.0 to an optimal value of 1.3 (lower was detrimental for permeability). Lanraplenib is a prime example of using a piperazine-oxetane as a more metabolically stable isostere of morpholine and of reducing planarity to improve drug-like properties.

In the development of danuglipron, a high-throughput screen identified pyrimidine-containing **6** as a weak GLP-1R agonist (glucagon-like peptide receptor 1) (Figure 4e).^36a^ In the final stages of the structure–activity relationship (SAR) study, the oxetane motif was introduced as a small polar head which increased potency without negatively impacting LogD and other physicochemical properties such as clearance and toxicity. Further notable changes include the introduction of the carboxylic acid, which reduced LogD and increased potency; the substitution of the fluoropyrimidine ring to a pyridine, which affected the dihedral angle between piperidine and the arene; and the exchange of a chloro for a cyano substituent, which improved metabolic stability. Danuglipron mimics the binding mode of peptide agonists to GLP-1R but circumvents the metabolic instability associated with peptidic therapeutics. In contrast to lotiglipron, a related oxetane-containing GLP-1R agonist that was withdrawn during phase I clinical trials due to undesirable drug–drug interactions, danuglipron showed no such concerns and was advanced to phase II trials.^36b^

GDC-0349 was developed during a late-stage optimization campaign to improve its predecessor’s (lead compound **7**) poor free plasma clearance and unfavorable time-dependent inhibition (TDI) of CYP enzymes, which can lead to issues on comedications and potential toxicity (Figure 4f).^37a^ It was speculated that the amino-pyrimidine functionality in compound **7** was metabolized oxidatively to an iminium quinone. Swapping the pyrimidine ring for alkyl groups on nitrogen indeed reduced CYP inhibition by >10-fold but suffered from high cardiac toxicity (hERG IC_50_ = 8.5 μM) related to the increased basicity of the tertiary alkylamine (p*K*_aH_ = 7.6). Introducing an oxetane substituent on nitrogen (GDC-0349) reduced p*K*_aH_ (5.0) and hERG inhibition (IC_50_ > 100 μM), while maintaining the low TDI. GDC-0349 was also highly selective against mTOR (mammalian target of rapamycin) over 266 other kinases and showed a 10-fold reduction in free plasma clearance compared to pyrimidine **7**.

**Figure 5:**
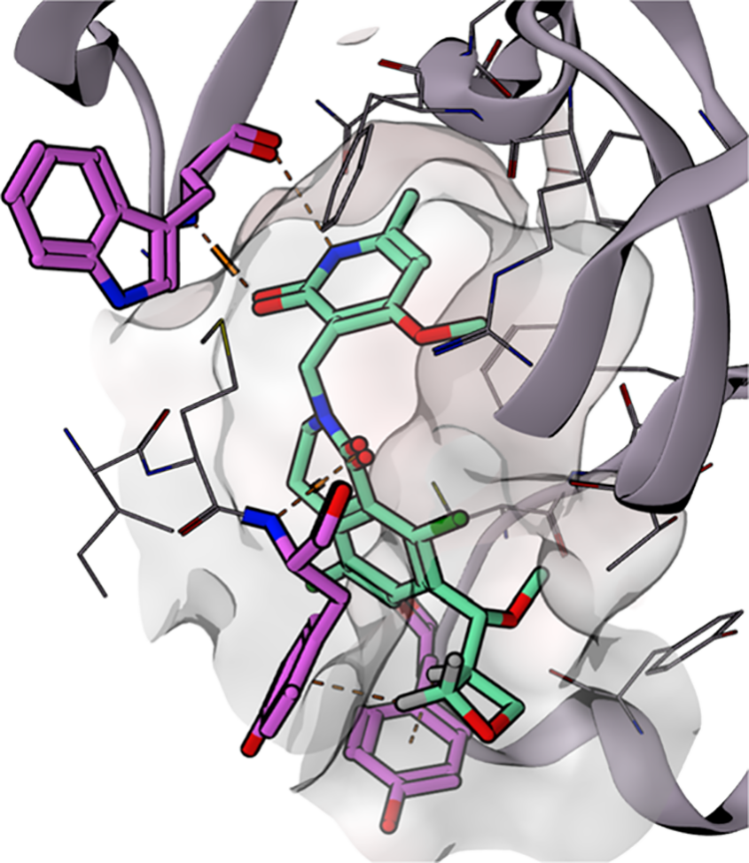
Cocrystal structure of oxetane **9** with EZH2 (see Figure 4g).^38^ Available under PDB code 4W2R (2.8 Å) and illustrated using MOE software.^40^

Lead compound **8** was a potent EZH2 (enhancer of zeste homologue 2) inhibitor but suffered from poor metabolic stability and insufficient solubility (Figure 4g).^38a^ It was hypothesized that substituting the dimethylisoxazole motif for an sp^3^ analogue would improve both properties by lowering LogD and increasing three-dimensionality. During the final SAR studies, a methoxymethyl-oxetane substituent (**9**) was introduced as a less lipophilic surrogate of a THF ring with an optimal LogD of 1.9 (lower was detrimental for permeability), with drastically improved metabolic and solubility properties, and a better fit into the protein pocket. The stereochemistry of the newly introduced stereogenic center α to oxetane was important for binding, with the (+)-(*R*) enantiomer (**9**) showing a 16-fold increase in potency compared to its enantiomer. A cocrystal structure of oxetane **9** in complex with EZH2 revealed the oxetane substituent to occupy a defined space in the protein cavity with two potential CH−π interactions between polarized oxetane CH groups and two tyrosine side chains (Figure 5).

The oxetane functionality was introduced at both early (fenebrutinib, ziresovir, lanraplenib, danuglipron) and late (GDC-0349, PF-06821497) stages of the synthetic sequences, and no instability issues were noted.^33−38^ The integrity of the oxetane ring was not compromised by conditions such as H_2_/Pd catalyst, NaBH_4_, (Boc)_2_O, DMAP, TsOH, aryllithium reagents, triazabicyclodecene (TBD), and KO^*t*^Bu.

### 2.3 Patents

**Figure 6:**
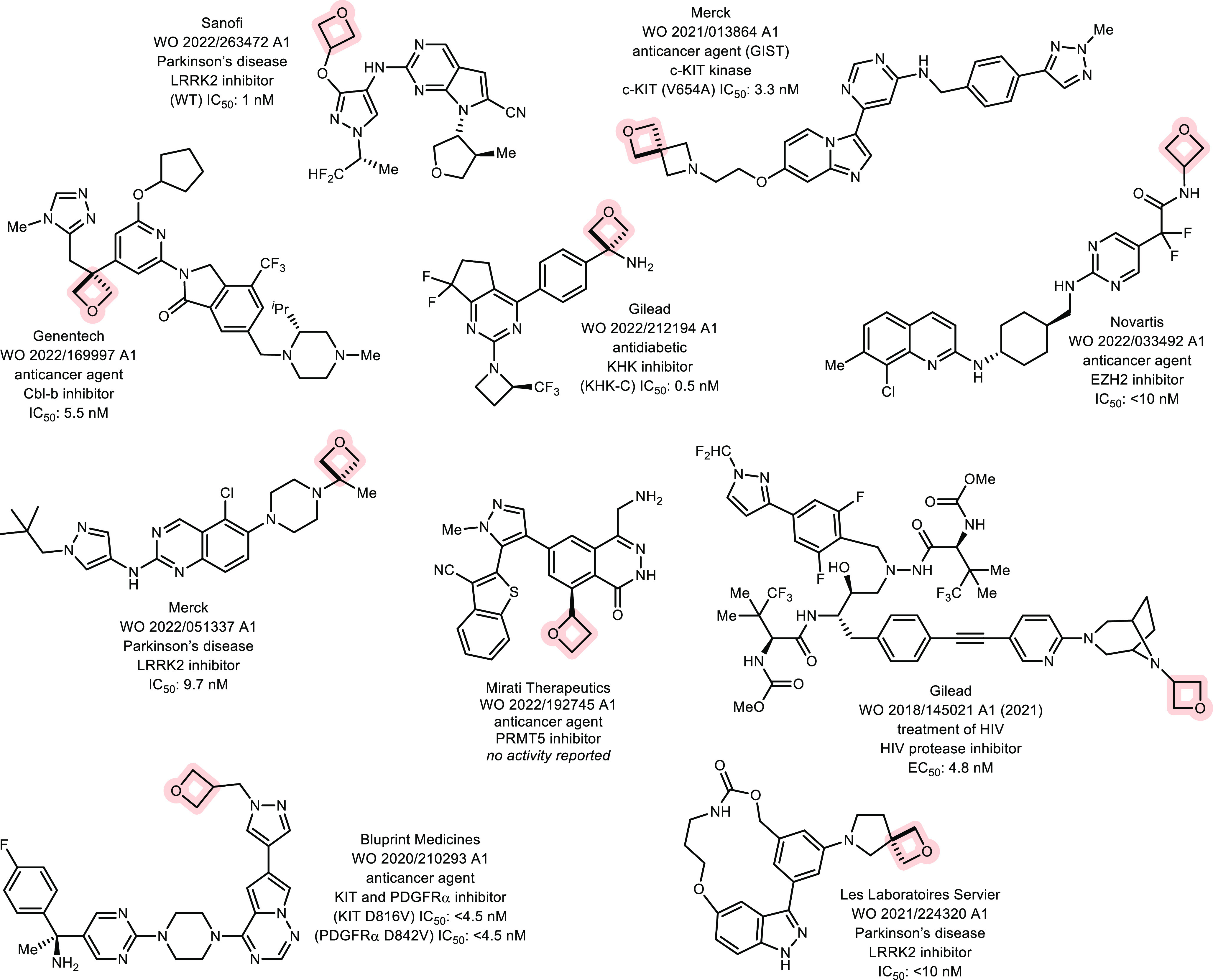
Selected examples of oxetanes in the patent literature (2017–2022).^49^ Cbl-b = Casitas B lymphoma-b; c-KIT, type III receptor tyrosine kinase; KHK = ketohexokinase; LRRK2, leucine rich repeat kinase 2; and PRMT5, protein arginine methyltransferase 5.

A considerable portion of the large number of patents filed with an oxetane structure (6007) is due, in part, to the now frequent inclusion of oxetane groups in claims to cover all the relevant IP space. Figure 6 shows selected examples from such patents, highlighting oxetane structures with varied substitution patterns.

### 2.4 Publication in Journals

**Figure 7:**
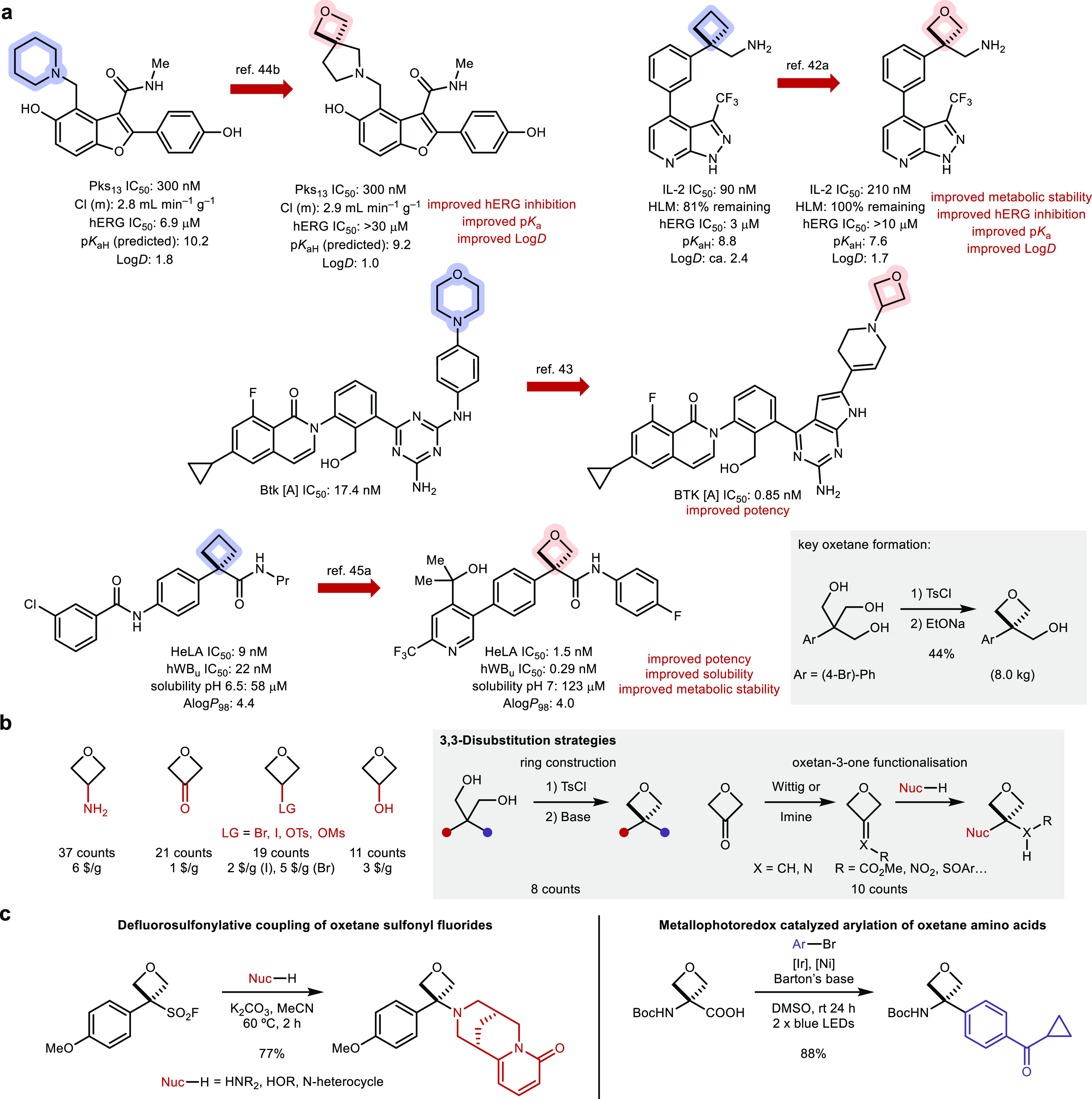
Oxetanes in drug discovery programs (2017–2022). (a) Selected examples in scientific articles. (b) Occurrence of the most popular oxetane building blocks used (from the refs in the Supporting Information). (c) Examples of recent methodologies developed for the synthesis of 3,3-disubstituted oxetanes.^28c,29a^ hWBu = human whole blood, unbound potency; IL-2, interleukin 2; and Pks_13_ = polyketide synthase 13.

In addition to the patent literature, oxetanes have appeared in over 100 peer-reviewed publications on medicinal chemistry campaigns between 2017–2022 (*vide supra* and the Supporting Information). Thirty-eight campaigns recognized an oxetane compound as the most promising structure, with the oxetane motif increasing solubility,^41^ metabolic stability,^41b,42^ permeability,^43^ reducing p*K*_aH_^44^ or LogD,^8a^^45^ or providing a better conformational fit into the desired target pocket (Figure 7a).^46^ Often, oxetane substitution was beneficial for several parameters simultaneously, as they can be intrinsically linked (e.g., LogD and metabolic stability or solubility). A popular approach was to incorporate an oxetane ring to increase steric bulk in a desired direction to improve affinity without raising LogD values to unacceptable levels.

Naturally, most campaigns that evaluated oxetanyl substituents did not choose an oxetane compound as the lead structure, as was found in 160 studies between 2017–2022 (Supporting Information). In most cases, this was due to higher potency of another scaffold and not because of unfavorable physicochemical properties of oxetane substituents. In fact, frequently, oxetane introduction had the desired physicochemical effect (e.g., lower p*K*_aH_ or LogD, increased stability, improved solubility) but did not provide sufficient bioactivity.^47^ Only in scattered examples was the oxetane analogue a chemical liability and was subsequently eliminated due to insufficient chemical stability, despite high potency and favorable PK properties.^48^ Despite oxetane’s potential to emulate the properties of carbonyl motifs, there were no medicinal applications as carbonyl isosteres, perhaps, due to the challenge to access the required 3,3-disubstitution on the oxetane ring and the dearth of methods to do so.

The source of oxetanes in medicinal chemistry campaigns provided another interesting analysis (Figure 7b). By far the most widely used oxetane building block is 3-amino-oxetane (37 counts), which served as a substrate in amide couplings, reductive amination, and S_N_Ar reactions, among others. This is followed by oxetan-3-one (21 counts), which was similarly involved in reductive amination reactions and also in organometallic carbonyl additions to yield substituted oxetanols. Further popular building blocks were oxetanes with a leaving group (LG) in the 3-position, involved in nucleophilic substitution reactions, and oxetan-3-ols, often used as nucleophiles.

There were two main strategies to access 3,3-disubstitution patterns: oxetane ring formation by intramolecular etherification (8 counts) and nucleophilic additions into oxetane alkylidenes/imines followed by functional group interconversions (10 counts). The two main drawbacks of these strategies are the use of reagents such as TsCl and strong bases (most often NaH) and the linear nature of the transformations: each analogue requires a distinct synthetic sequence. The commercial availability of 3-monosubstituted oxetane building blocks by far exceeds that of 2-substituted or 3,3-disubstituted examples, which is reflected in their increased appearance in medicinal chemistry programs (Figure 7b). The increased availability appears to have influenced oxetane substitution patterns in active pharmaceutical ingredients (APIs), which are primarily 3-mono-substituted (see Figure 4). 3,3-Disubstituted oxetanes are potentially more attractive (e.g., as isosteres or more stable derivatives) but still suffer from a higher synthetic burden and a lack of useful methods for their incorporation, demanding more progress from the synthetic community. As such, several promising new approaches have emerged in recent years such as the defluorosulfonylative coupling of oxetane sulfonyl fluorides with nucleophiles^28c^ or the metallophotoredox catalyzed decarboxylative arylation of oxetane amino acids (Figure 7c).^29a^

## 3 Conclusions

The oxetane scaffold has transformed from an academic curiosity to a valuable motif for contemporary drug discovery. Pioneering studies from Carreira and co-workers with collaborators at Roche on oxetanes as bioisosteres of *gem*-dimethyl and carbonyl groups initiated an “oxetane rush” in the medicinal chemistry community that was for some met with early disenchantment due to the potential chemical instability and synthetic intractability of the oxetane ring. Follow-up studies demonstrated stability to be strongly linked to the substitution pattern on oxetane, with 3,3-disubstitution being most stable. Advances in the synthesis and pharmacological evaluation of substituted oxetane compounds have been notable, improving the general understanding of the effect of the oxetane motif to drug-relevant properties and facilitating the inclusion of oxetane rings into medicinal chemistry programs. Synthetic and medicinal research in academia and industry in the last 20 years has uncovered the many potential advantages of including oxetanes into a drug compound, but also the pitfalls and challenges. Here we provide a summary and perspective on these endeavors as the following take-home messages.

### 3.1 Potential Benefits of the Oxetane Motif

(1) The inductive electron-withdrawing effect of the oxetane ring reduces the p*K*_aH_ of adjacent basic functionality by ca. 2.7 units α, 1.9 units β, 0.7 units γ, and 0.3 units δ. Tactical placement of an oxetane ring can be used to reduce or remove issues associated with the basicity of a drug compound.^16^ (2) The three tetrahedral, sp^3^-hybridized carbon atoms impart the oxetane ring with increased three-dimensionality. This conformational effect can lead to an increase in the aqueous solubility of the target compound and also gives access to unexplored chemical space. (3) The small size and marked polarity of the oxetane scaffold can be used to block metabolically labile sites and/or introduce steric bulk without significantly increasing molecular weight or lipophilicity. (4) The structural and H-bond acceptor similarities of oxetanes with carbonyls render oxetane motifs potential bioisosteres of the latter, which could be useful to circumvent carbonyl-specific enzymatic degradation, improve aqueous solubility, or access new IP space. (5) The moderate ring strain associated with the oxetane ring could be leveraged to direct the metabolism of APIs to be cleared by mEH instead of CYP enzymes, which could be useful to prevent undesired drug–drug interactions that can cause liver toxicity on comedication.^23,36b^

### 3.2 Pitfalls, Challenges, And Future Directions

(1) Despite the advances in new methodologies, accessing the desired substitution on oxetane is still a considerable synthetic challenge. Although construction of the oxetane ring at a late stage can be an effective solution (e.g., Figure 7b), this approach is linear and leaves little room for the rapid generation of analogues. (2) Related to point 1, the choice of oxetane building blocks is limited. This constraint has influenced substitution patterns of oxetane structures in drug compounds (Figure 7b). Most notable has been the use of 3-amino-oxetane and oxetan-3-one building blocks, with the latter often being used in reductive amination reactions. New useful oxetane building blocks would increase the diversity of oxetane substitution in investigational compounds. In this vein, we recently developed oxetane sulfonyl fluoride reagents that allow the coupling of 3-aryl-oxetane fragments with diverse nucleophiles.^28^ Complimentarily, Terrett and Huestis developed a decarboxylative strategy to couple oxetane amino acid building blocks to abundant aryl halides.^29a^ (3) Oxetane rings can be unstable toward ring-opening, particularly under acidic conditions or high temperatures, and it is challenging to predict stability of a given oxetane substitution pattern. A general rule of thumb is that 3,3-disubstituted examples are more stable than other substitution patterns, but stability is nevertheless not guaranteed and could become a metabolic and/or chemical liability *in vivo*. For example, 3,3-disubstituted oxetanes with an internal nucleophile (e.g., alcohol or amine functionality) more readily ring-open under acidic conditions.^24,25^ (4) Related to point 3, the potential instability of oxetane rings under harsh reaction conditions might become an issue for multistep large scale synthesis. In general, oxetane moieties were introduced only during the final stages of drug development to remediate problematic physicochemical properties such as insufficient solubility or metabolic stability (see section 2.2). Hence, methods that install oxetane rings at a late stage would be valuable to circumvent degradative pitfalls (see, e.g., Figure 7c).^28c,29a^ There is also little data on oxetane synthesis on process scales, and it is unclear how stable oxetanes would be to potential local hot spots in the reactor. One example was reported by Li and Sloman (Merck) who synthesized a functionalized oxetane ring by C–O bond formation on an 8.0 kg scale (Figure 7a, boxed). (5) As insinuated in points 3 and 4, the data sets available that report on relevant properties of oxetane compounds are limited, which hampers data-based predictions. For example, the metabolic fate of oxetane compounds (i.e., clearance by mEH vs CYPs) could not be correlated to intrinsic properties (e.g., p*K*_a_, LogD, partial charges) on the (small) set of compounds tested and was deemed substrate specific. Additionally, as seen with the recent withdrawal of oxetane-containing lotiglipron (see section 2.2), drug–drug interactions are still challenging to predict and can lead to drug attrition during clinical development.^36b^ More experimental data on medicinally relevant properties of oxetane compounds will improve the general understanding of the effect of introducing an oxetane ring and increase the quality of data-based predictive models.

Despite the absence of a fully synthetic oxetane-containing drug on the market (i.e., not from the taxane family), the increased appearance of oxetanes in clinical candidates, investigational compounds, and scientific reports leaves no doubt that they will soon be a mainstay of commercial drugs.

## Supporting Information Available

Further details on the occurrence of thietanes as well as a comprehensive list of references that report an oxetane structure in a drug discovery campaign between 2017–2022 (PDF)

## Abbreviations Used

AML: acute myeloid leukemia
API: active pharmaceutical ingredient
Btk: Bruton’s tyrosine kinase
cAMP: cyclic adenosine monophosphate
Cbl-b: Casitas B lymphoma-b
CC_50_: half maximal cytotoxic concentration
Cl: clearance
Cl_int_: intrinsic clearance
c-KIT: type III receptor tyrosine kinase
CYP: cytochrome P450
DMAP: 4-dimethylaminopyridine
EC_50_: half maximal effective concentration
EZH2: enhancer of zest homologue 2
FDA: Food and Drug Administration
FLT3: fms like tyrosine kinase 3
GIST: gastrointestinal stromal tumor
GLP-1R: glucagon-like peptide receptor 1
hERG: human Ether-à-go-go-Related Gene
HIV: human immunodeficiency virus
HLM: human liver microsomes
HTS: high-throughput screening
hWBu: human whole blood, unbound potency
IC_50_: half maximal inhibitory concentration
IL-2: interleukin 2
IP: intellectual property
KHK: ketohexokinase
LG: leaving group
LMN: lupus membranous nephropathy
LRRK2: leucine rich repeat kinase 2
mEH: microsomal epoxide hydrolase
MOE: molecular operating environment
MS: multiple sclerosis
mTOR: mammalian target of rapamycin
PDB: Protein Data Bank
PDGFRα: platelet-derived growth factor receptor
PK: pharmacokinetic
Pks_13_: polyketide synthase 13
PrCP: prolylcarboxypeptidase
PRMT5: protein arginine methyltransferase 5
RSV: respiratory syncytial virus
SAR: structure–activity relationship
SCLC: small cell lung cancer
S_N_Ar: nucleophilic aromatic substitution
SYK: spleen tyrosine kinase
TBD: triazabicyclodecene
TDI: time-dependent inhibition
THF: tetrahydrofuran
TI: therapeutic index
*V*_ss_: volume of distribution
WHO: World Health Organization
Y641N: mutant form of EZH2
