# Supplementary material for: Oxetanes in Drug Discovery Campaigns
Source: J Med Chem. 2023 Sep 7;66(18):12697–709. doi: 10.1021/acs.jmedchem.3c01101 (PMC10544023; doi:10.1021/acs.jmedchem.3c01101)
Supplement: Supplementary file 1 — jm3c01101_si_001.pdf [file jm3c01101_si_001.pdf]

SUPPORTING INFORMATION

Oxetanes in Drug Discovery Campaigns

Juan J. Rojas,[a] and James, A. Bull*,[a]

[a] Department of Chemistry, Imperial College London, Molecular Sciences Research Hub, White City Campus, Wood Lane, London W12 0BZ, UK.

*E-mail: j.bull@imperial.ac.uk

Table of contents

Appearance of Thietane Rings in the Literature S2
Complete List of References of Oxetanes in Medicinal Chemistry Campaigns S3–S13

Appearance of Thietane Rings in the Literature

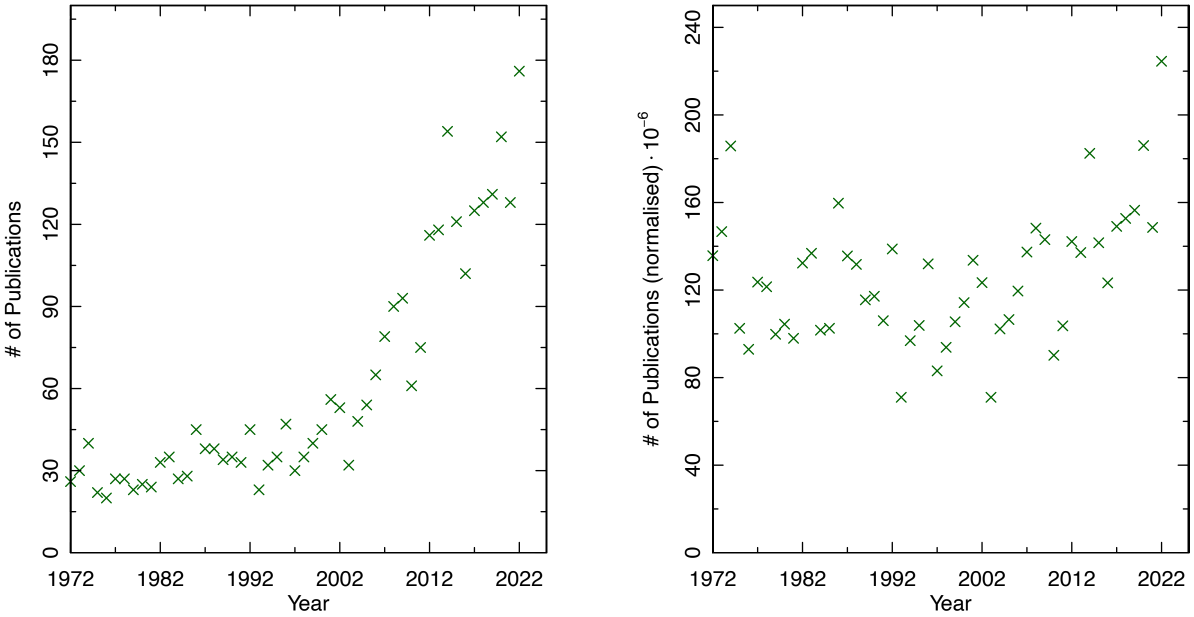

Figure S1. Number of publications that include a thietane ring between 1972 and 2022. Left: Absolute number of publications. Right: normalised against the total number of publications recorded per year. Values obtained from a Scifinder search in January 2023. Publications included that report: biological study, therapeutic use, pharmacological activity, preparation, synthetic preparation, biological study (unclassified), pharmacokinetics, biological use (unclassified), agricultural use, biosynthetic preparation and natural product occurrence. The analysis also included the sulfoxide and sulfone oxidation states.

Complete List of References of Oxetanes in Medicinal Chemistry Campaigns

A comprehensive list of references of all medicinal chemistry campaigns that included an oxetane structure between 2017–2022 in scientific articles is listed below ordered by year:

2017

Low, J. D.; Bartberger, M. D.; Chen, K.; Cheng, Y.; Fielden, M. R.; Gore, V.; Hickman, D.; Liu, Q.; Allen Sickmier, E.; Vargas, H. M.; Werner, J.; White, R. D.; Whittington, D. A.; Wood, S.; Minatti, A. E. *Medchemcomm* **2017**, *8*, 1196–1206. Eastman, K. J.; Parcella, K.; Yeung, K.-S.; Grant-Young, K. A.; Zhu, J.; Wang, T.; Zhang, Z.; Yin, Z.; Beno, B. R.; Sheriff, S.; Kish, K.; Tredup, J.; Jardel, A. G.; Halan, V.; Ghosh, K.; Parker, D.; Mosure, K.; Fang, H.; Wang, Y.-K.; Lemm, J.; Zhuo, X.; Hanumegowda, U.; Rigat, K.; Donoso, M.; Tuttle, M.; Zvyaga, T.; Haarhoff, Z.; Meanwell, N. A.; Soars, M. G.; Roberts, S. B.; Kadow, J. F. *Medchemcomm* **2017**, *8*, 796–806. Feng, S.; Li, C.; Chen, D.; Zheng, X.; Yun, H.; Gao, L.; Shen, H. C. *Eur. J. Med. Chem.* **2017**, *138*, 1147–1157. Wang, X.; Barbosa, J.; Blomgren, P.; Bremer, M. C.; Chen, J.; Crawford, J. J.; Deng, W.; Dong, L.; Eigenbrot, C.; Gallion, S.; Hau, J.; Hu, H.; Johnson, A. R.; Katewa, A.; Kropf, J. E.; Lee, S. H.; Liu, L.; Lubach, J. W.; Macaluso, J.; Maciejewski, P.; Mitchell, S. A.; Ortwine, D. F.; DiPaolo, J.; Reif, K.; Scheerens, H.; Schmitt, A.; Wong, H.; Xiong, J.-M.; Xu, J.; Zhao, Z.; Zhou, F.; Currie, K. S.; Young, W. B. *ACS Med. Chem. Lett.* **2017**, *8*, 608–613. Millan, D. S.; Kayser-Bricker, K. J.; Martin, M. W.; Talbot, A. C.; Schiller, S. E. R.; Herbertz, T.; Williams, G. L.; Luke, G. P.; Hubbs, S.; Alvarez Morales, M. A.; Cardillo, D.; Troccolo, P.; Mendes, R. L.; McKinnon, C. *ACS Med. Chem. Lett.* **2017**, *8*, 847–852. Llona-Minguez, S.; Höglund, A.; Ghassemian, A.; Desroses, M.; Calderón-Montaño, J. M.; Burgos Morón, E.; Valerie, N. C. K.; Wiita, E.; Almlöf, I.; Koolmeister, T.; Mateus, A.; Cazares-Körner, C.; Sanjiv, K.; Homan, E.; Loseva, O.; Baranczewski, P.; Darabi, M.; Mehdizadeh, A.; Fayezi, S.; Jemth, A.-S.; Warpman Berglund, U.; Sigmundsson, K.; Lundbäck, T.; Jenmalm Jensen, A.; Artursson, P.; Scobie, M.; Helleday, T. *J. Med. Chem.* **2017**, *60*, 4279–4292. Burks, H. E.; Abrams, T.; Kirby, C. A.; Baird, J.; Fekete, A.; Hamann, L. G.; Kim, S.; Lombardo, F.; Loo, A.; Lubicka, D.; Macchi, K.; McDonnell, D. P.; Mishina, Y.; Norris, J. D.; Nunez, J.; Saran, C.; Sun, Y.; Thomsen, N. M.; Wang, C.; Wang, J.; Peukert, S. *J. Med. Chem.* **2017**, *60*, 2790–2818. Nordqvist, A.; O'Mahony, G.; Fridén-Saxin, M.; Fredenwall, M.; Hogner, A.; Granberg, K. L.; Aagaard, A.; Bäckström, S.; Gunnarsson, A.; Kaminski, T.; Xue, Y.; Dellsén, A.; Hansson, E.; Hansson, P.; Ivarsson, I.; Karlsson, U.; Bamberg, K.; Hermansson, M.; Georgsson, J.; Lindmark, B.; Edman, K. *ChemMedChem* **2017**, *12*, 50–65. Gibson, T. S.; Johnson, B.; Fanjul, A.; Halkowycz, P.; Dougan, D. R.; Cole, D.; Swann, S. *Bioorg. Med. Chem. Lett.* **2017**, *27*, 1709–1713. Wang, L.; Pratt, J. K.; Soltwedel, T.; Sheppard, G. S.; Fidanze, S. D.; Liu, D.; Hasvold, L. A.; Mantei, R. A.; Holms, J. H.; McClellan, W. J.; Wendt, M. D.; Wada, C.; Frey, R.; Hansen, T. M.; Hubbard, R.; Park, C. H.; Li, L.; Magoc, T. J.; Albert, D. H.; Lin, X.; Warder, S. E.; Kovar, P.; Huang, X.; Wilcox, D.; Wang, R.; Rajaraman, G.; Petros, A. M.; Hutchins, C. W.; Panchal, S. C.; Sun, C.; Elmore, S. W.; Shen, Y.; Kati, W. M.; McDaniel, K. F. *J. Med. Chem.* **2017**, *60*, 3828–3850. Riggs, J. R.; Nagy, M.; Elsner, J.; Erdman, P.; Cashion, D.; Robinson, D.; Harris, R.; Huang, D.; Tehrani, L.; Deyanat-Yazdi, G.; Narla, R. K.; Peng, X.; Tran, T.; Barnes, L.; Miller, T.; Katz, J.; Tang, Y.; Chen, M.; Moghaddam, M. F.; Bahmanyar, S.; Pagarigan, B.; Delker, S.; LeBrun, L.; Chamberlain, P. P.; Calabrese, A.; Canan, S. S.; Leftheris, K.; Zhu, D.; Boylan, J. F. *J. Med. Chem.* **2017**, *60*, 8989–9002. Siu, T.; Brubaker, J.; Fuller, P.; Torres, L.; Zeng, H.; Close, J.; Mampreian, D. M.; Shi, F.; Liu, D.; Fradera, X.; Johnson, K.; Bays, N.; Kadic, E.; He, F.; Goldenblatt, P.; Shaffer, L.; Patel, S. B.; Lesburg, C. A.; Alpert, C.; Dorosh, L.; Deshmukh, S. V.; Yu, H.; Klappenbach, J.; Elwood, F.; Dinsmore, C. J.; Fernandez, R.; Moy, L.; Young, J. R. *J. Med. Chem.* **2017**, *60*, 9676–9690. Bezençon, O.; Heidmann, B.; Siegrist, R.; Stamm, S.; Richard, S.; Pozzi, D.; Corminboeuf, O.; Roch, C.; Kessler, M.; Ertel, E. A.; Reymond, I.; Pfeifer, T.; de Kanter, R.; Toeroek-Schafroth, M.; Moccia, L. G.; Mawet, J.; Moon, R.; Rey, M.; Capeleto, B.; Fournier, E. *J. Med. Chem.* **2017**, *60*, 9769–9789. Boga, S. B.; Alhassan, A.-B.; Liu, J.; Guiadeen, D.; Krikorian, A.; Gao, X.; Wang, J.; Yu, Y.; Anand, R.; Liu, S.; Yang, C.; Wu, H.; Cai, J.; Zhu, H.; Desai, J.; Maloney, K.; Gao, Y.-D.; Fischmann, T. O.; Presland, J.; Mansueto, M.; Xu, Z.; Leccese, E.; Knemeyer, I.; Garlisi, C. G.; Bays, N.; Stivers, P.; Brandish, P. E.; Hicks, A.; Cooper, A.; Kim, R. M.; Kozlowski, J. A. *Bioorg. Med. Chem. Lett.* **2017**, *27*, 3939–3943. Patel, S.; Meilandt, W. J.; Erickson, R. I.; Chen, J.; Deshmukh, G.; Estrada, A. A.; Fuji, R. N.; Gibbons, P.; Gustafson, A.; Harris, S. F.; Imperio, J.; Liu, W.; Liu, X.; Liu, Y.; Lyssikatos, J. P.; Ma, C.; Yin, J.; Lewcock, J. W.; Siu, M. *J. Med. Chem.* **2017**, *60*, 8083–8102.

2018

Poce, G.; Cocozza, M.; Alfonso, S.; Consalvi, S.; Venditti, G.; Fernandez-Menendez, R.; Bates, R. H.; Barros Aguirre, D.; Ballell, L.; De Logu, A.; Vistoli, G.; Biava, M. *Eur. J. Med. Chem.* **2018**, *145*, 539–550. Cheong, J. E.; Zaffagni, M.; Chung, I.; Xu, Y.; Wang, Y.; Jernigan, F. E.; Zetter, B. R.; Sun, L. *Eur. J. Med. Chem.* **2018**, *144*, 372–385. Vazquez, M. L.; Kaila, N.; Strohbach, J. W.; Trzupek, J. D.; Brown, M. F.; Flanagan, M. E.; Mitton-Fry, M. J.; Johnson, T. A.; TenBrink, R. E.; Arnold, E. P.; Basak, A.; Heasley, S. E.; Kwon, S.; Langille, J.; Parikh, M. D.; Griffin, S. H.; Casavant, J. M.; Duclos, B. A.; Fenwick, A. E.; Harris, T. M.; Han, S.; Caspers, N.; Dowty, M. E.; Yang, X.; Banker, M. E.; Hegen, M.; Symanowicz, P. T.; Li, L.; Wang, L.; Lin, T. H.; Jussif, J.; Clark, J. D.; Telliez, J.-B.; Robinson, R. P.; Unwalla, R. *J. Med. Chem.* **2018**, *61*, 1130–1152. Kung, P.-P.; Bingham, P.; Brooun, A.; Collins, M.; Deng, Y.-L.; Dinh, D.; Fan, C.; Gajiwala, K. S.; Grantner, R.; Gukasyan, H. J.; Hu, W.; Huang, B.; Kania, R.; Kephart, S. E.; Krivacic, C.; Kumpf, R. A.; Khamphavong, P.; Kraus, M.; Liu, W.; Maegley, K. A.; Nguyen, L.; Ren, S.; Richter, D.; Rollins, R. A.; Sach, N.; Sharma, S.; Sherrill, J.; Spangler, J.; Stewart, A. E.; Sutton, S.; Uryu, S.; Verhelle, D.; Wang, H.; Wang, S.; Wythes, M.; Xin, S.; Yamazaki, S.; Zhu, H.; Zhu, J.; Zehnder, L.; Edwards, M. *J. Med. Chem.* **2018**, *61*, 650–665. Soares, P.; Gadd, M. S.; Frost, J.; Galdeano, C.; Ellis, L.; Epemolu, O.; Rocha, S.; Read, K. D.; Ciulli, A. *J. Med. Chem.* **2018**, *61*, 599–618. Crawford, J. J.; Johnson, A. R.; Misner, D. L.; Belmont, L. D.; Castanedo, G.; Choy, R.; Coraggio, M.; Dong, L.; Eigenbrot, C.; Erickson, R.; Ghilardi, N.; Hau, J.; Katewa, A.; Kohli, P. B.; Lee, W.; Lubach, J. W.; McKenzie, B. S.; Ortwine, D. F.; Schutt, L.; Tay, S.; Wei, B.; Reif, K.; Liu, L.; Wong, H.; Young, W. B. *J. Med. Chem.* **2018**, *61*, 2227–2245. Pu, S.-Y.; Wouters, R.; Schor, S.; Rozenski, J.; Barouch-Bentov, R.; Prugar, L. I.; O'Brien, C. M.; Brannan, J. M.; Dye, J. M.; Herdewijn, P.; De Jonghe, S.; Einav, S. *J. Med. Chem.* **2018**, *61*, 6178–6192. Procopiou, P. A.; Anderson, N. A.; Barrett, J.; Barrett, T. N.; Crawford, M. H. J.; Fallon, B. J.; Hancock, A. P.; Le, J.; Lemma, S.; Marshall, R. P.; Morrell, J.; Pritchard, J. M.; Rowedder, J. E.; Saklatvala, P.; Slack, R. J.; Sollis, S. L.; Suckling, C. J.; Thorp, L. R.; Vitulli, G.; Macdonald, S. J. F. *J. Med. Chem.* **2018**, *61*, 8417–8443. Rogacki, M. K.; Pitta, E.; Balabon, O.; Huss, S.; Lopez-Roman, E. M.; Argyrou, A.; Blanco-Ruano, D.; Cacho, M.; Vande Velde, C. M. L.; Augustyns, K.; Ballell, L.; Barros, D.; Bates, R. H.; Cunningham, F.; Van der Veken, P. *J. Med. Chem.* **2018**, *61*, 11221–11249. LaPorte, M. G.; Burnett, J. C.; Colombo, R.; Bulfer, S. L.; Alverez, C.; Chou, T.-F.; Neitz, R. J.; Green, N.; Moore, W. J.; Yue, Z.; Li, S.; Arkin, M. R.; Wipf, P.; Huryn, D. M. *ACS Med. Chem. Lett.* **2018**, *9*, 1075–1081. Akama, T.; Zhang, Y.-K.; Freund, Y. R.; Berry, P.; Lee, J.; Easom, E. E.; Jacobs, R. T.; Plattner, J. J.; Witty, M. J.; Peter, R.; Rowan, T. G.; Gillingwater, K.; Brun, R.; Nare, B.; Mercer, L.; Xu, M.; Wang, J.; Liang, H. *Bioorg. Med. Chem. Lett.* **2018**, *28*, 6–10. Chowdhury, S.; Owens, K. N.; Herr, R. J.; Jiang, Q.; Chen, X.; Johnson, G.; Groppi, V. E.; Raible, D. W.; Rubel, E. W.; Simon, J. A. *J. Med. Chem.* **2018**, *61*, 84–97. Chevillard, F.; Rimmer, H.; Betti, C.; Pardon, E.; Ballet, S.; van Hilten, N.; Steyaert, J.; Diederich, W. E.; Kolb, P. *J. Med. Chem.* **2018**, *61*, 1118–1129. Rahm, F.; Viklund, J.; Trésaugues, L.; Ellermann, M.; Giese, A.; Ericsson, U.; Forsblom, R.; Ginman, T.; Günther, J.; Hallberg, K.; Lindström, J.; Persson, L. B.; Silvander, C.; Talagas, A.; Díaz-Sáez, L.; Fedorov, O.; Huber, K. V. M.; Panagakou, I.; Siejka, P.; Gorjánácz, M.; Bauser, M.; Andersson, M. *J. Med. Chem.* **2018**, *61*, 2533–2551. Lu, H.; Yang, T.; Xu, Z.; Lin, X.; Ding, Q.; Zhang, Y.; Cai, X.; Dong, K.; Gong, S.; Zhang, W.; Patel, M.; Copley, R. C. B.; Xiang, J.; Guan, X.; Wren, P.; Ren, F. *J. Med. Chem.* **2018**, *61*, 2518–2532. Amato, G. S.; Manke, A.; Vasukuttan, V.; Wiethe, R. W.; Snyder, R. W.; Runyon, S. P.; Maitra, R. *Bioorg. Med. Chem.* **2018**, *26*, 4518–4531. Vandyck, K.; Rombouts, G.; Stoops, B.; Tahri, A.; Vos, A.; Verschueren, W.; Wu, Y.; Yang, J.; Hou, F.; Huang, B.; Vergauwen, K.; Dehertogh, P.; Berke, J. M.; Raboisson, P. *J. Med. Chem.* **2018**, *61*, 6247–6260. Pike, K. G.; Barlaam, B.; Cadogan, E.; Campbell, A.; Chen, Y.; Colclough, N.; Davies, N. L.; De-Almeida, C.; Degorce, S. L.; Didelot, M.; Dishington, A.; Ducray, R.; Durant, S. T.; Hassall, L. A.; Holmes, J.; Hughes, G. D.; MacFaul, P. A.; Mulholland, K. R.; McGuire, T. M.; Ouvry, G.; Pass, M.; Robb, G.; Stratton, N.; Wang, Z.; Wilson, J.; Zhai, B.; Zhao, K.; Al-Huniti, N. *J. Med. Chem.* **2018**, *61*, 3823–3841. Giroud, M.; Kuhn, B.; Saint-Auret, S.; Kuratli, C.; Martin, R. E.; Schuler, F.; Diederich, F.; Kaiser, M.; Brun, R.; Schirmeister, T.; Haap, W. *J. Med. Chem.* **2018**, *61*, 3370–3388. Huddle, B. C.; Grimley, E.; Buchman, C. D.; Chtcherbinine, M.; Debnath, B.; Mehta, P.; Yang, K.; Morgan, C. A.; Li, S.; Felton, J.; Sun, D.; Mehta, G.; Neamati, N.; Buckanovich, R. J.; Hurley, T. D.; Larsen, S. D. *J. Med. Chem.* **2018**, *61*, 8754–8773. Liu, X.; Zhang, Y.; Huang, W.; Tan, W.; Zhang, A. *Bioorg. Med. Chem.* **2018**, *26*, 443–454.

2019

Cao, H.; Zhu, G.; Sun, L.; Chen, G.; Ma, X.; Luo, X.; Zhu, J. *Eur. J. Med. Chem.* **2019**, *183*, 111694. Chen, P.; Zhang, D.; Li, M.; Wu, Q.; Lam, Y. P. Y.; Guo, Y.; Chen, C.; Bai, N.; Malhotra, S.; Li, W.; O'Connor, P. B.; Fu, H. *Eur. J. Med. Chem.* **2019**, *183*, 111722. Zhu, Y.; Sun, N.; Yu, M.; Guo, H.; Xie, Q.; Wang, Y. *Eur. J. Med. Chem.* **2019**, *182*, 111589. Chen, S.; Guo, W.; Liu, X.; Sun, P.; Wang, Y.; Ding, C.; Meng, L.; Zhang, A. *Eur. J. Med. Chem.* **2019**, *179*, 38–55. Zhang, C.; Pei, H.; He, J.; Zhu, J.; Li, W.; Niu, T.; Xiang, M.; Chen, L. *Eur. J. Med. Chem.* **2019**, *169*, 121–143. Bagal, S. K.; Omoto, K.; Blakemore, D. C.; Bungay, P. J.; Bilsland, J. G.; Clarke, P. J.; Corbett, M. S.; Cronin, C. N.; Cui, J. J.; Dias, R.; Flanagan, N. J.; Greasley, S. E.; Grimley, R.; Johnson, E.; Fengas, D.; Kitching, L.; Kraus, M. L.; McAlpine, I.; Nagata, A.; Waldron, G. J.; Warmus, J. S. *J. Med. Chem.* **2019**, *62*, 247–265. Guerrero, M.; Urbano, M.; Kim, E.-K.; Gamo, A. M.; Riley, S.; Abgaryan, L.; Leaf, N.; Van Orden, L. J.; Brown, S. J.; Xie, J. Y.; Porreca, F.; Cameron, M. D.; Rosen, H.; Roberts, E. *J. Med. Chem.* **2019**, *62*, 1761–1780. Caille, S.; Cui, S.; Faul, M. M.; Mennen, S. M.; Tedrow, J. S.; Walker, S. D. *J. Org. Chem.* **2019**, *84*, 4583–4603. Kong, D.; Guo, S.; Yang, Y.; Guo, B.; Xie, X.; Hu, W. *Bioorg. Med. Chem. Lett.* **2019**, *29*, 848–852. Wang, H.-L.; Andrews, K. L.; Booker, S. K.; Canon, J.; Cee, V. J.; Chavez, F.; Chen, Y.; Eastwood, H.; Guerrero, N.; Herberich, B.; Hickman, D.; Lanman, B. A.; Laszlo, J.; Lee, M. R.; Lipford, J. R.; Mattson, B.; Mohr, C.; Nguyen, Y.; Norman, M. H.; Pettus, L. H.; Powers, D.; Reed, A. B.; Rex, K.; Sastri, C.; Tamayo, N.; Wang, P.; Winston, J. T.; Wu, B.; Wu, Q.; Wu, T.; Wurz, R. P.; Xu, Y.; Zhou, Y.; Tasker, A. S. *J. Med. Chem.* **2019**, *62*, 1523–1540. Shi, C.; Zhang, Y.; Wang, T.; Lu, W.; Zhang, S.; Guo, B.; Chen, Q.; Luo, C.; Zhou, X.; Yang, Y. *J. Med. Chem.* **2019**, *62*, 2950–2973. Aguilar, A.; Zheng, K.; Xu, T.; Xu, S.; Huang, L.; Fernandez-Salas, E.; Liu, L.; Bernard, D.; Harvey, K. P.; Foster, C.; McEachern, D.; Stuckey, J.; Chinnaswamy, K.; Delproposto, J.; Kampf, J. W.; Wang, S. *J. Med. Chem.* **2019**, *62*, 6015–6034. Alen, J.; Schade, M.; Wagener, M.; Christian, F.; Nordhoff, S.; Merla, B.; Dunkern, T. R.; Bahrenberg, G.; Ratcliffe, P. *J. Med. Chem.* **2019**, *62*, 6391–6397. Fleau, C.; Padilla, A.; Miguel-Siles, J.; Quesada-Campos, M. T.; Saiz-Nicolas, I.; Cotillo, I.; Cantizani Perez, J.; Tarleton, R. L.; Marco, M.; Courtemanche, G. *J. Med. Chem.* **2019**, *62*, 10362–10375. Werner, S.; Mesch, S.; Hillig, R. C.; ter Laak, A.; Klint, J.; Neagoe, I.; Laux-Biehlmann, A.; Dahllöf, H.; Bräuer, N.; Puetter, V.; Nubbemeyer, R.; Schulz, S.; Bairlein, M.; Zollner, T. M.; Steinmeyer, A. *J. Med. Chem.* **2019**, *62*, 11194–11217. Dai, W.; Samanta, S.; Xue, D.; Petrunak, E. M.; Stuckey, J. A.; Han, Y.; Sun, D.; Wu, Y.; Neamati, N. *J. Med. Chem.* **2019**, *62*, 3068–3087. Toledo-Sherman, L.; Breccia, P.; Cachope, R.; Bate, J. R.; Angulo-Herrera, I.; Wishart, G.; Matthews, K. L.; Martin, S. L.; Cox, H. C.; McAllister, G.; Penrose, S. D.; Vater, H.; Esmieu, W.; Van de Poël, A.; Van de Bospoort, R.; Strijbosch, A.; Lamers, M.; Leonard, P.; Jarvis, R. E.; Blackaby, W.; Barnes, K.; Eznarriaga, M.; Dowler, S.; Smith, G. D.; Fischer, D. F.; Lazari, O.; Yates, D.; Rose, M.; Jang, S.-W.; Muñoz-Sanjuan, I.; Dominguez, C. *J. Med. Chem.* **2019**, *62*, 2988–3008. Bell, M.; Foley, D.; Naylor, C.; Wood, G.; Robinson, C.; Riley, J.; Epemolu, O.; Ellis, L.; Scullion, P.; Shishikura, Y.; Osuna-Cabello, M.; Ferguson, L.; Pinto, E.; Fletcher, D.; Katz, E.; McLean, W. H. I.; Wyatt, P.; Read, K. D.; Woodland, A. *ACS Med. Chem. Lett.* **2019**, *10*, 341–347. Meinig, J. M.; Ferrara, S. J.; Banerji, T.; Banerji, T.; Sanford-Crane, H. S.; Bourdette, D.; Scanlan, T. S. *ACS Med. Chem. Lett.* **2019**, *10*, 111–116. Felts, A. S.; Bollinger, K. A.; Brassard, C. J.; Rodriguez, A. L.; Morrison, R. D.; Scott Daniels, J.; Blobaum, A. L.; Niswender, C. M.; Jones, C. K.; Jeffrey Conn, P.; Emmitte, K. A.; Lindsley, C. W. *Bioorg. Med. Chem. Lett.* **2019**, *29*, 47–50. Kaieda, A.; Takahashi, M.; Fukuda, H.; Okamoto, R.; Morimoto, S.; Gotoh, M.; Miyazaki, T.; Hori, Y.; Unno, S.; Kawamoto, T.; Tanaka, T.; Itono, S.; Takagi, T.; Sugimoto, H.; Okada, K.; Lane, W.; Sang, B.; Saikatendu, K.; Matsunaga, S.; Miwatashi, S. *ChemMedChem* **2019**, *14*, 2093–2101. Meng, W.; Adam, L. P.; Behnia, K.; Zhao, L.; Yang, R.; Kopcho, L. M.; Locke, G. A.; Taylor, D. S.; Yin, X.; Wexler, R. R.; Finlay, H. *Bioorg. Med. Chem. Lett.* **2019**, *29*, 126673. Amato, G.; Wiethe, R.; Manke, A.; Vasukuttan, V.; Snyder, R.; Runyon, S.; Maitra, R. *Bioorg. Med. Chem.* **2019**, *27*, 3632–3649. Mazur, M.; Dymek, B.; Koralewski, R.; Sklepkiewicz, P.; Olejniczak, S.; Mazurkiewicz, M.; Piotrowicz, M.; Salamon, M.; Jędrzejczak, K.; Zagozdzon, A.; Czestkowski, W.; Matyszewski, K.; Borek, B.; Bartoszewicz, A.; Pluta, E.; Rymaszewska, A.; Mozga, W.; Stefaniak, F.; Dobrzański, P.; Dzwonek, K.; Golab, J.; Golebiowski, A.; Olczak, J. *J. Med. Chem.* **2019**, *62*, 7126–7145. Owen, R. M.; Blakemore, D. C.; Cao, L.; Flanagan, N.; Fish, R.; Gibson, K. R.; Gurrell, R.; Huh, C. W.; Kammonen, J.; Mortimer-Cassen, E.; Nickolls, S.; Omoto, K.; Owen, D. R.; Pike, A.; Pryde, D. C.; Reynolds, D.; Roeloffs, R.; Rose, C. R.; Stead, C.; Takeuchi, M.; Warmus, J. S.; Watson, C. *J. Med. Chem.* **2019**, *62*, 5773–5796. Velcicky, J.; Mathison, C. J. N.; Nikulin, V.; Pflieger, D.; Epple, R.; Azimioara, M.; Cow, C.; Michellys, P.-Y.; Rigollier, P.; Beisner, D. R.; Bodendorf, U.;

Guerini, D.; Liu, B.; Wen, B.; Zaharevitz, S.; Brandl, T. *ACS Med. Chem. Lett.* **2019**, *10*, 887–892. Cioffi, C. L.; Racz, B.; Varadi, A.; Freeman, E. E.; Conlon, M. P.; Chen, P.; Zhu, L.; Kitchen, D. B.; Barnes, K. D.; Martin, W. H.; Pearson, P. G.; Johnson, G.; Blaner, W. S.; Petrukhin, K. *J. Med. Chem.* **2019**, *62*, 5470–5500. Fushimi, M.; Fujimori, I.; Wakabayashi, T.; Hasui, T.; Kawakita, Y.; Imamura, K.; Kato, T.; Murakami, M.; Ishii, T.; Kikko, Y.; Kasahara, M.; Nakatani, A.; Hiura, Y.; Miyamoto, M.; Saikatendu, K.; Zou, H.; Lane, S. W.; Lawson, J. D.; Imoto, H. *J. Med. Chem.* **2019**, *62*, 4915–4935. Kahl, D. J.; Hutchings, K. M.; Lisabeth, E. M.; Haak, A. J.; Leipprandt, J. R.; Dexheimer, T.; Khanna, D.; Tsou, P.-S.; Campbell, P. L.; Fox, D. A.; Wen, B.; Sun, D.; Bailie, M.; Neubig, R. R.; Larsen, S. D. *J. Med. Chem.* **2019**, *62*, 4350–4369. Riggs, J. R.; Elsner, J.; Cashion, D.; Robinson, D.; Tehrani, L.; Nagy, M.; Fultz, K. E.; Krishna Narla, R.; Peng, X.; Tran, T.; Kulkarni, A.; Bahmanyar, S.; Condroski, K.; Pagarigan, B.; Fenalti, G.; LeBrun, L.; Leftheris, K.; Zhu, D.; Boylan, J. F. *J. Med. Chem.* **2019**, *62*, 4401–4410. Lin, S.; Jin, J.; Liu, Y.; Tian, H.; Zhang, Y.; Fu, R.; Zhang, J.; Wang, M.; Du, T.; Ji, M.; Wu, D.; Zhang, K.; Sheng, L.; Li, Y.; Chen, X.; Xu, H. *J. Med. Chem.* **2019**, *62*, 8873–8879. Mullarky, E.; Xu, J.; Robin, A. D.; Huggins, D. J.; Jennings, A.; Noguchi, N.; Olland, A.; Lakshminarasimhan, D.; Miller, M.; Tomita, D.; Michino, M.; Su, T.; Zhang, G.; Stamford, A. W.; Meinke, P. T.; Kargman, S.; Cantley, L. C. *Bioorg. Med. Chem. Lett.* **2019**, *29*, 2503–2510. Collier, P. N.; Twin, H. C.; Knegtel, R. M. A.; Boyall, D.; Brenchley, G.; Davis, C. J.; Keily, S.; Mak, C.; Miller, A.; Pierard, F.; Settimo, L.; Bolton, C. M.; Chiu, P.; Curnock, A.; Doyle, E.; Tanner, A. J.; Jimenez, J.-M. *ACS Med. Chem. Lett.* **2019**, *10*, 1134–1139.

2020

Xu, G.; Zhang, Y.; Wang, H.; Guo, Z.; Wang, X.; Li, X.; Chang, S.; Sun, T.; Yu, Z.; Xu, T.; Zhao, L.; Wang, Y.; Yu, W. *Eur. J. Med. Chem.* **2020**, *198*, 112354. Zhu, F.; Wang, Y.; Du, Q.; Ge, W.; Li, Z.; Wang, X.; Fu, C.; Luo, L.; Tian, S.; Ma, H.; Zheng, J.; Zhang, Y.; Sun, X.; He, S.; Zhang, X. *Eur. J. Med. Chem.* **2020**, *187*, 111914. Zhu, C.; Li, X.; Zhao, B.; Peng, W.; Li, W.; Fu, W. *Eur. J. Med. Chem.* **2020**, *193*, 112214.Khanna, A.; Côté, A.; Arora, S.; Moine, L.; Gehling, V. S.; Brenneman, J.; Cantone, N.; Stuckey, J. I.; Apte, S.; Ramakrishnan, A.; Bruderek, K.; Bradley, W. D.; Audia, J. E.; Cummings, R. T.; Sims, R. J.; Trojer, P.; Levell, J. R. *ACS Med. Chem. Lett.* **2020**, *11*, 1205–1212. Wellaway, C. R.; Amans, D.; Bamborough, P.; Barnett, H.; Bit, R. A.; Brown, J. A.; Carlson, N. R.; Chung, C.; Cooper, A. W. J.; Craggs, P. D.; Davis, R. P.; Dean, T. W.; Evans, J. P.; Gordon, L.; Harada, I. L.; Hirst, D. J.; Humphreys, P. G.; Jones, K. L.; Lewis, A. J.; Lindon, M. J.; Lugo, D.; Mahmood, M.; McCleary, S.; Medeiros, P.; Mitchell, D. J.; O'Sullivan, M.; Le Gall, A.; Patel, V. K.; Patten, C.; Poole, D. L.; Shah, R. R.; Smith, J. E.; Stafford, K. A. J.; Thomas, P. J.; Vimal, M.; Wall, I. D.; Watson, R. J.; Wellaway, N.; Yao, G.; Prinjha, R. K. *J. Med. Chem.* **2020**, *63*, 714–746. Blomgren, P.; Chandrasekhar, J.; Di Paolo, J. A.; Fung, W.; Geng, G.; Ip, C.; Jones, R.; Kropf, J. E.; Lansdon, E. B.; Lee, S.; Lo, J. R.; Mitchell, S. A.; Murray, B.; Pohlmeyer, C.; Schmitt, A.; Suekawa-Pirrone, K.; Wise, S.; Xiong, J.-M.; Xu, J.; Yu, H.; Zhao, Z.; Currie, K. S. *ACS Med. Chem. Lett.* **2020**, *11*, 506–513. Panchaud, P.; Surivet, J.-P.; Diethelm, S.; Blumstein, A.-C.; Gauvin, J.-C.; Jacob, L.; Masse, F.; Mathieu, G.; Mirre, A.; Schmitt, C.; Enderlin-Paput, M.; Lange, R.; Gnerre, C.; Seeland, S.; Herrmann, C.; Locher, H. H.; Seiler, P.; Ritz, D.; Rueedi, G. *J. Med. Chem.* **2020**, *63*, 88–102. Kwiatkowski, J.; Liu, B.; Pang, S.; Ahmad, N. H. B.; Wang, G.; Poulsen, A.; Yang, H.; Poh, Y. R.; Tee, D. H. Y.; Ong, E.; Retna, P.; Dinie, N.; Kwek, P.; Wee, J. L. K.; Manoharan, V.; Low, C. B.; Seah, P. G.; Pendharkar, V.; Sangthongpitag, K.; Joy, J.; Baburajendran, N.; Jansson, A. E.; Nacro, K.; Hill, J.; Keller, T. H.; Hung, A. W. *J. Med. Chem.* **2020**, *63*, 621–637. Shirai, F.; Mizutani, A.; Yashiroda, Y.; Tsumura, T.; Kano, Y.; Muramatsu, Y.; Chikada, T.; Yuki, H.; Niwa, H.; Sato, S.; Washizuka, K.; Koda, Y.; Mazaki, Y.; Jang, M.-K.; Yoshida, H.; Nagamori, A.; Okue, M.; Watanabe, T.; Kitamura, K.; Shitara, E.; Honma, T.; Umehara, T.; Shirouzu, M.; Fukami, T.; Seimiya, H.; Yoshida, M.; Koyama, H. *J. Med. Chem.* **2020**, *63*, 4183–4204. Leger, P. R.; Hu, D. X.; Biannic, B.; Bui, M.; Han, X.; Karbarz, E.; Maung, J.; Okano, A.; Osipov, M.; Shibuya, G. M.; Young, K.; Higgs, C.; Abraham, B.; Bradford, D.; Cho, C.; Colas, C.; Jacobson, S.; Ohol, Y. M.; Pookot, D.; Rana, P.; Sanchez, J.; Shah, N.; Sun, M.; Wong, S.; Brockstedt, D. G.; Kassner, P. D.; Schwarz, J. B.; Wustrow, D. J. *J. Med. Chem.* **2020**, *63*, 5398–5420. Vendeville, S.; Tahri, A.; Hu, L.; Demin, S.; Cooymans, L.; Vos, A.; Kwanten, L.; Van den Berg, J.; Battles, M. B.; McLellan, J. S.; Koul, A.; Raboisson, P.; Roymans, D.; Jonckers, T. H. M. *J. Med. Chem.* **2020**, *63*, 8046–8058. Wang, C.; Pei, Y.; Wang, L.; Li, S.; Jiang, C.; Tan, X.; Dong, Y.; Xiang, Y.; Ma, Y.; Liu, G. *J. Med. Chem.* **2020**, *63*, 6066–6089. Li, Y.; Zhao, J.; Gutgesell, L. M.; Shen, Z.; Ratia, K.; Dye, K.; Dubrovskyi, O.; Zhao, H.; Huang, F.; Tonetti, D. A.; Thatcher, G. R. J.; Xiong, R. *J. Med. Chem.* **2020**, *63*, 7186–7210. Rodríguez Sarmiento, R. M.; Bissantz, C.; Bylund, J.; Limberg, A.; Neidhart, W.; Jakob-Roetne, R.; Wang, L.; Baumann, K. *J. Med. Chem.* **2020**, *63*, 8534–8553. Shen, Y.; Gao, G.; Yu, X.; Kim, H.; Wang, L.; Xie, L.; Schwarz, M.; Chen, X.; Guccione, E.; Liu, J.; Bedford, M. T.; Jin, J. *J. Med. Chem.* **2020**, *63*, 9977–9989. Furukawa, H.; Miyamoto, Y.; Hirata, Y.; Watanabe, K.; Hitomi, Y.; Yoshitomi, Y.; Aida, J.; Noguchi, N.; Takakura, N.; Takami, K.; Miwatashi, S.; Hirozane, Y.; Hamada, T.; Ito, R.; Ookawara, M.; Moritoh, Y.; Watanabe, M.; Maekawa, T. *J. Med. Chem.* **2020**, *63*, 10352–10379. Chen, J.; Zhou, Y.; Dong, X.; Liu, L.; Bai, L.; McEachern, D.; Przybranowski, S.; Yang, C.-Y.; Stuckey, J.; Li, X.; Wen, B.; Zhao, T.; Sun, S.; Sun, D.; Jiao, L.; Jing, Y.; Guo, M.; Yang, D.; Wang, S. *J. Med. Chem.* **2020**, *63*, 13994–14016. Hügle, M.; Regenass, P.; Warstat, R.; Hau, M.; Schmidtkunz, K.; Lucas, X.; Wohlwend, D.; Einsle, O.; Jung, M.; Breit, B.; Günther, S. *J. Med. Chem.* **2020**, *63*, 15603–15620. Martin, M. C.; Zeng, G.; Yu, J.; Schiltz, G. E. *J. Med. Chem.* **2020**, *63*, 15344–15370. Crawford, J. J.; Lee, W.; Johnson, A. R.; Delatorre, K. J.; Chen, J.; Eigenbrot, C.; Heidmann, J.; Kakiuchi-Kiyota, S.; Katewa, A.; Kiefer, J. R.; Liu, L.; Lubach, J. W.; Misner, D.; Purkey, H.; Reif, K.; Vogt, J.; Wong, H.; Yu, C.; Young, W. B. *ACS Med. Chem. Lett.* **2020**, *11*, 1588–1597. Gehling, V. S.; McGrath, J. P.; Duplessis, M.; Khanna, A.; Brucelle, F.; Vaswani, R. G.; Côté, A.; Stuckey, J.; Watson, V.; Cummings, R. T.; Balasubramanian, S.; Iyer, P.; Sawant, P.; Good, A. C.; Albrecht, B. K.; Harmange, J.-C.; Audia, J. E.; Bellon, S. F.; Trojer, P.; Levell, J. R. *ACS Med. Chem. Lett.* **2020**, *11*, 1213–1220. Grandjean, J.-M. M.; Jiu, A. Y.; West, J. W.; Aoyagi, A.; Droege, D. G.; Elepano, M.; Hirasawa, M.; Hirouchi, M.; Murakami, R.; Lee, J.; Sasaki, K.; Hirano, S.; Ohyama, T.; Tang, B. C.; Vaz, R. J.; Inoue, M.; Olson, S. H.; Prusiner, S. B.; Conrad, J.; Paras, N.

A. *ACS Med. Chem. Lett.* **2020**, *11*, 127–132. Methot, J. L.; Achab, A.; Christopher, M.; Zhou, H.; McGowan, M. A.; Trotter, B. W.; Fradera, X.; Lesburg, C. A.; Goldenblatt, P.; Hill, A.; Chen, D.; Otte, K. M.; Augustin, M.; Shah, S.; Katz, J. D. *ACS Med. Chem. Lett.* **2020**, *11*, 2461–2469. Yamada, Y.; Takashima, H.; Walmsley, D. L.; Ushiyama, F.; Matsuda, Y.; Kanazawa, H.; Yamaguchi-Sasaki, T.; Tanaka-Yamamoto, N.; Yamagishi, J.; Kurimoto-Tsuruta, R.; Ogata, Y.; Ohtake, N.; Angove, H.; Baker, L.; Harris, R.; Macias, A.; Robertson, A.; Surgenor, A.; Watanabe, H.; Nakano, K.; Mima, M.; Iwamoto, K.; Okada, A.; Takata, I.; Hitaka, K.; Tanaka, A.; Fujita, K.; Sugiyama, H.; Hubbard, R. E. *J. Med. Chem.* **2020**, *63*, 14805–14820. Larsen, J.; Lambert, M.; Pettersson, H.; Vifian, T.; Larsen, M.; Ollerstam, A.; Hegardt, P.; Eskilsson, C.; Laursen, S.; Soehoel, A.; Skak-Nielsen, T.; Hansen, L. M.; Knudsen, N. Ø.; Eirefelt, S.; Sørensen, M. D.; Stilou, T. G.; Nielsen, S. F. *J. Med. Chem.* **2020**, *63*, 14502–14521. Degorce, S. L.; Aagaard, A.; Anjum, R.; Cumming, I. A.; Diène, C. R.; Fallan, C.; Johnson, T.; Leuchowius, K.-J.; Orton, A. L.; Pearson, S.; Robb, G. R.; Rosen, A.; Scarfe, G. B.; Scott, J. S.; Smith, J. M.; Steward, O. R.; Terstiege, I.; Tucker, M. J.; Turner, P.; Wilkinson, S. D.; Wrigley, G. L.; Xue, Y. *Bioorg. Med. Chem.* **2020**, *28*, 115815. Durand-Réville, T. F.; Comita-Prevoir, J.; Zhang, J.; Wu, X.; May-Dracka, T. L.; Romero, J. A. C.; Wu, F.; Chen, A.; Shapiro, A. B.; Carter, N. M.; McLeod, S. M.; Giacobbe, R. A.; Verheijen, J. C.; Lahiri, S. D.; Sacco, M. D.; Chen, Y.; O'Donnell, J. P.; Miller, A. A.; Mueller, J. P.; Tommasi, R. A. *J. Med. Chem.* **2020**, *63*, 12511–12525. Zhan, W.; Singh, P. K.; Ban, Y.; Qing, X.; Ah Kioon, M. D.; Fan, H.; Zhao, Q.; Wang, R.; Sukenick, G.; Salmon, J.; Warren, J. D.; Ma, X.; Barrat, F. J.; Nathan, C. F.; Lin, G. *J. Med. Chem.* **2020**, *63*, 13103–13123. Dong, J.; Huang, J.; Zhou, J.; Tan, Y.; Jin, J.; Tan, X.; Wang, B.; Yu, T.; Wu, C.; Chen, S.; Wang, T.-L. *ACS Med. Chem. Lett.* **2020**, *11*, 1463–1469. Klug, D. M.; Tschiegg, L.; Diaz, R.; Rojas-Barros, D.; Perez-Moreno, G.; Ceballos, G.; García-Hernández, R.; Martinez-Martinez, M. S.; Manzano, P.; Ruiz, L. M.; Caffrey, C. R.; Gamarro, F.; Pacanowska, D. G.; Ferrins, L.; Navarro, M.; Pollastri, M. P. *J. Med. Chem.* **2020**, *63*, 2527–2546. Zhu, C.; Li, X.; Peng, W.; Fu, W. *Molecules* **2020**, *25*, 5078. Yaragani, M.; Yadlapalli, P.; Raghavan, S.; Ayyadurai, N.; Chinnusamy, S.; Mandava, V. B. R.; Kottapalli, R. P. *J. Chem. Sci.* **2020**, *132*, 136. Mlakar, L.; Lane, J.; Takihara, T.; Lim, C.; Sprachman, M. M.; Lloyd, K. R.; Wipf, P.; Feghali-Bostwick, C. *ACS Med. Chem. Lett.* **2020**, *11*, 2312–2317. Henley, Z. A.; Amour, A.; Barton, N.; Bantscheff, M.; Bergamini, G.; Bertrand, S. M.; Convery, M.; Down, K.; Dümpelfeld, B.; Edwards, C. D.; Grandi, P.; Gore, P. M.; Keeling, S.; Livia, S.; Mallett, D.; Maxwell, A.; Price, M.; Rau, C.; Reinhard, F. B. M.; Rowedder, J.; Rowland, P.; Taylor, J. A.; Thomas, D. A.; Hessel, E. M.; Hamblin, J. N. *J. Med. Chem.* **2020**, *63*, 638–655. White, C.; McGowan, M. A.; Zhou, H.; Sciammetta, N.; Fradera, X.; Lim, J.; Joshi, E. M.; Andrews, C.; Nickbarg, E. B.; Cowley, P.; Trewick, S.; Augustin, M.; von Köenig, K.; Lesburg, C. A.; Otte, K.; Knemeyer, I.; Woo, H.; Yu, W.; Cheng, M.; Spacciapoli, P.; Geda, P.; Song, X.; Smotrov, N.; Curran, P.; Heo, M. R.; Abeywickrema, P.; Miller, J. R.; Bennett, D. J.; Han, Y. *ACS Med. Chem. Lett.* **2020**, *11*, 550–557.

2021

Pan, C.; Nie, W.; Wang, J.; Du, J.; Pan, Z.; Gao, J.; Lu, Y.; Che, J.; Zhu, H.; Dai, H.; Chen, B.; He, Q.; Dong, X. *Eur. J. Med. Chem.* **2021**, *225*, 113794. Zhao, L.; Yin, W.; Sun, Y.; Sun, N.; Tian, L.; Zheng, Y.; Zhang, C.; Zhao, S.; Su, X.; Zhao, D.; Cheng, M. *Eur. J. Med. Chem.* **2021**, *224*, 113715. Wu, C.-J.; Wu, J.-Q.; Hu, Y.; Pu, S.; Lin, Y.; Zeng, Z.; Hu, J.; Chen, W.-H. *Eur. J. Med. Chem.* **2021**, *223*, 113629. Ran, K.; Zeng, J.; Wan, G.; He, X.; Feng, Z.; Xiang, W.; Wei, W.; Hu, X.; Wang, N.; Liu, Z.; Yu, L. *Eur. J. Med. Chem.* **2021**, *220*, 113499. Wu, S.; Xu, C.; Xia, K.; Lin, Y.; Tian, S.; Ma, H.; Ji, Y.; Zhu, F.; He, S.; Zhang, X. *Eur. J. Med. Chem.* **2021**, *217*, 113327. Maciuszek, M.; Ortega-Gomez, A.; Maas, S. L.; Perretti, M.; Merritt, A.; Soehnlein, O.; Chapman, T. M. *Eur. J. Med. Chem.* **2021**, *214*, 113194. Xia, Y.; Yu, M.; Zhao, Y.; Xia, L.; Huang, Y.; Sun, N.; Song, M.; Guo, H.; Zhang, Y.; Zhu, D.; Xie, Q.; Wang, Y. *Eur. J. Med. Chem.* **2021**, *211*, 113013. Gulati, A.; Yeung, C. S.; Lapointe, B.; Kattar, S. D.; Gunaydin, H.; Scott, J. D.; Childers, K. K.; Methot, J. L.; Simov, V.; Kurukulasuriya, R.; Pio, B.; Morriello, G. J.; Liu, P.; Tang, H.; Neelamkavil, S.; Wood, H. B.; Rada, V. L.; Ardolino, M. J.; Yan, X. C.; Palte, R.; Otte, K.; Faltus, R.; Woodhouse, J.; Hegde, L. G.; Ciaccio, P.; Minnihan, E. C.; DiMauro, E. F.; Fell, M. J.; Fuller, P. H.; Ellis, J. M. *RSC Med. Chem.* **2021**, *12*, 1164–1173. Akao, Y.; Canan, S.; Cao, Y.; Condroski, K.; Engkvist, O.; Itono, S.; Kaki, R.; Kimura, C.; Kogej, T.; Nagaoka, K.; Naito, A.; Nakai, H.; Pairaudeau, G.; Radu, C.; Roberts, I.; Shimada, M.; Shum, D.; Watanabe, N.; Xie, H.; Yonezawa, S.; Yoshida, O.; Yoshida, R.; Mowbray, C.; Perry, B. *RSC Med. Chem.* **2021**, *12*, 384–393. Zhang, G.; Sheng, L.; Hegde, P.; Li, Y.; Aldrich, C. C. *Med. Chem. Res.* **2021**, *30*, 449–458. Huddle, B. C.; Grimley, E.; Chtcherbinine, M.; Buchman, C. D.; Takahashi, C.; Debnath, B.; McGonigal, S. C.; Mao, S.; Li, S.; Felton, J.; Pan, S.; Wen, B.; Sun, D.; Neamati, N.; Buckanovich, R. J.; Hurley, T. D.; Larsen, S. D. *Eur. J. Med. Chem.* **2021**, *211*, 113060. Jung, Y.-H.; Salmaso, V.; Wen, Z.; Bennett, J. M.; Phung, N. B.; Lieberman, D. I.; Gopinatth, V.; Randle, J. C. R.; Chen, Z.; Salvemini, D.; Karcz, T. P.; Cook, D. N.; Jacobson, K. A. *J. Med. Chem.* **2021**, *64*, 5099–5122. Safina, B. S.; McKerrall, S. J.; Sun, S.; Chen, C. A.; Chowdhury, S.; Jia, Q.; Li, J.; Zenova, A. Y.; Andrez, J. C.; Bankar, G.; Bergeron, P.; Chang, J. H.; Chang, E.; Chen, J.; Dean, R.; Decker, S. M.; Dipasquale, A.; Focken, T.; Hemeon, I.; Khakh, K.; Kim, A.; Kwan, R.; Lindgren, A.; Lin, S.; Maher, J.; Mezeyova, J.; Misner, D.; Nelkenbrecher, K.; Pang, J.; Reese, R.; Shields, S. D.; Sojo, L.; Sheng, T.; Verschoof, H.; Waldbrook, M.; Wilson, M. S.; Xie, Z.; Young, C.; Zabka, T. S.; Hackos, D. H.; Ortwine, D. F.; White, A. D.; Johnson, J. P.; Robinette, C. L.; Dehnhardt, C. M.; Cohen, C. J.; Sutherlin, D. P. *J. Med. Chem.* **2021**, *64*, 2953–2966. Safina, B. S.; McKerrall, S. J.; Sun, S.; Chen, C.-A.; Chowdhury, S.; Jia, Q.; Li, J.; Zenova, A. Y.; Andrez, J.-C.; Bankar, G.; Bergeron, P.; Chang, J. H.; Chang, E.; Chen, J.; Dean, R.; Decker, S. M.; DiPasquale, A.; Focken, T.; Hemeon, I.; Khakh, K.; Kim, A.; Kwan, R.; Lindgren, A.; Lin, S.; Maher, J.; Mezeyova, J.; Misner, D.; Nelkenbrecher, K.; Pang, J.; Reese, R.; Shields, S. D.; Sojo, L.; Sheng, T.; Verschoof, H.; Waldbrook, M.; Wilson, M. S.; Xie, Z.; Young, C.; Zabka, T. S.; Hackos, D. H.; Ortwine, D. F.; White, A. D.; Johnson, J. P.; Robinette, C. L.; Dehnhardt, C. M.; Cohen, C. J.; Sutherlin, D. P. *J. Med. Chem.* **2021**, *64*, 2953–2966. Wu, Y.; Wang, Q.; Jiang, M.-Y.; Huang, Y.-Y.; Zhu, Z.; Han, C.; Tian, Y.-J.; Zhang, B.; Luo, H.-B. *J. Med. Chem.* **2021**, *64*, 9537–9549. Liang, J.; Zbieg, J. R.; Blake, R. A.; Chang, J. H.; Daly, S.; DiPasquale, A. G.; Friedman, L. S.; Gelzleichter, T.; Gill, M.; Giltnane, J. M.; Goodacre, S.; Guan, J.; Hartman, S. J.; Ingalla, E. R.; Kategaya, L.; Kiefer, J. R.; Kleinheinz, T.; Labadie, S. S.; Lai, T.; Li, J.; Liao, J.; Liu, Z.; Mody, V.; McLean, N.; Metcalfe, C.; Nannini, M. A.; Oeh, J.; O'Rourke, M. G.; Ortwine, D. F.; Ran, Y.; Ray, N. C.; Roussel, F.; Sambrone, A.; Sampath, D.; Schutt, L. K.; Vinogradova, M.; Wai, J.; Wang, T.; Wertz, I. E.; White, J. R.; Yeap, S. K.; Young, A.; Zhang, B.; Zheng, X.; Zhou, W.; Zhong, Y.; Wang, X. *J. Med. Chem.* **2021**, *64*, 11841–11856. Rianjongdee, F.; Atkinson, S. J.; Chung, C.; Grandi, P.; Gray, J. R. J.; Kaushansky, L. J.; Medeiros, P.; Messenger, C.; Phillipou, A.; Preston, A.; Prinjha, R. K.; Rioja, I.; Satz, A. L.; Taylor, S.; Wall, I. D.; Watson, R. J.; Yao, G.; Demont, E. H. *J. Med. Chem.* **2021**, *64*, 10806–10833. Zhang, Z.; Ghosh, A.; Connolly, P. J.; King, P.; Wilde, T.; Wang, J.; Dong, Y.; Li, X.; Liao, D.; Chen, H.; Tian, G.; Suarez, J.; Bonnette, W. G.; Pande, V.; Diloreto, K. A.; Shi, Y.; Patel, S.; Pietrak, B.; Szewczuk, L.; Sensenhauser, C.; Dallas, S.; Edwards, J. P.; Bachman, K. E.; Evans, D. C. *J. Med. Chem.* **2021**, *64*, 11570–11596. Aicher, T. D.; Van Huis, C. A.; Hurd, A. R.; Skalitzky, D. J.; Taylor, C. B.; Beleh, O. M.; Glick, G.; Toogood, P. L.; Yang, B.; Zheng, T.; Huo, C.; Gao, J.; Qiao, C.; Tian, X.; Zhang, J.; Demock, K.; Hao, L.-Y.; Lesch, C. A.; Morgan, R. W.; Moisan, J.; Wang, Y.; Scatina, J.; Paulos, C. M.; Zou, W.; Carter, L. L.; Hu, X. *J. Med. Chem.* **2021**, *64*, 13410–13428. Röhm, S.; Berger, B.-T.; Schröder, M.; Chatterjee, D.; Mathea, S.; Joerger, A. C.; Pinkas, D. M.; Bufton, J. C.; Tjaden, A.; Kovooru, L.; Kudolo, M.; Pohl, C.; Bullock, A. N.; Müller, S.; Laufer, S.; Knapp, S. *J. Med. Chem.*

2021, *64*, 13451–13474. Chen, L.; Su, M.; Jin, Q.; Wang, W.; Wang, C.-G.; Assani, I.; Wang, M.-X.; Zhao, S.-F.; Lv, S.-M.; Wang, J.-W.; Sun, B.; Li, Y.; Liao, Z.-X. *J. Med. Chem.* **2021**, *64*, 16106–16131. Come, J. H.; Senter, T. J.; Clark, M. P.; Court, J. J.; Gale-Day, Z.; Gu, W.; Krueger, E.; Liang, J.; Morris, M.; Nanthakumar, S.; O'Dowd, H.; Maltais, F.; Iyer, G.; Andreassi, J.; Boucher, C.; Considine, T.; Moody, C. S.; Taylor, W.; Mohanty, A. K.; Huang, Y.; Zuccola, H.; Coll, J.; Bonanno, K. C.; Gagnon, K. J.; Gan, L.; Lu, F.; Gao, H.; Chakilam, A.; Engtrakul, J.; Song, B.; Crawford, D.; Doyle, E.; Kramer, T.; Vought, B.; Phillips, J.; Kemper, R.; Sanders, M.; Swett, R.; Furey, B.; Winquist, R.; Bunnage, M. E.; Jackson, K. L.; Charifson, P. S.; Magavi, S. S. *J. Med. Chem.* **2021**, *64*, 17753–17776. Lillich, F. F.; Willems, S.; Ni, X.; Kilu, W.; Borkowsky, C.; Brodsky, M.; Kramer, J. S.; Brunst, S.; Hernandez-Olmos, V.; Heering, J.; Schierle, S.; Kestner, R.-I.; Mayser, F. M.; Helmstädter, M.; Göbel, T.; Weizel, L.; Namgaladze, D.; Kaiser, A.; Steinhilber, D.; Pfeilschifter, W.; Kahnt, A. S.; Proschak, A.; Chaikuad, A.; Knapp, S.; Merk, D.; Proschak, E. *J. Med. Chem.* **2021**, *64*, 17259–17276. Li, D.; Deng, Y.; Achab, A.; Bharathan, I.; Hopkins, B. A.; Yu, W.; Zhang, H.; Sanyal, S.; Pu, Q.; Zhou, H.; Liu, K.; Lim, J.; Fradera, X.; Lesburg, C. A.; Lammens, A.; Martinot, T. A.; Cohen, R. D.; Doty, A. C.; Ferguson, H.; Nickbarg, E. B.; Cheng, M.; Spacciapoli, P.; Geda, P.; Song, X.; Smotrov, N.; Abeywickrema, P.; Andrews, C.; Chamberlin, C.; Mabrouk, O.; Curran, P.; Richards, M.; Saradjian, P.; Miller, J. R.; Knemeyer, I.; Otte, K. M.; Vincent, S.; Sciammetta, N.; Pasternak, A.; Bennett, D. J.; Han, Y. *ACS Med. Chem. Lett.* **2021**, *12*, 389–396. Narayanan, S.; Wang, S.; Vasukuttan, V.; Vyas Devambatla, R. K.; Dai, D.; Jin, C.; Snyder, R.; Laudermilk, L.; Runyon, S. P.; Maitra, R. *J. Med. Chem.* **2021**, *64*, 3006–3025. Josa-Culleré, L.; Cogswell, T.; Georgiou, I.; Jay-Smith, M.; Jackson, T.; Bataille, C.; Davies, S.; Vyas, P.; Milne, T.; Wynne, G.; Russell, A. *Molecules* **2021**, *26*, 6648. Liu, Q.; Luo, Y.; Li, Z.; Chen, C.; Fang, L. *Bioorg. Med. Chem.* **2021**, *36*, 116094. Palmer, M. J.; Deng, X.; Watts, S.; Krilov, G.; Gerasyuto, A.; Kokkonda, S.; El Mazouni, F.; White, J.; White, K. L.; Striepen, J.; Bath, J.; Schindler, K. A.; Yeo, T.; Shackleford, D. M.; Mok, S.; Deni, I.; Lawong, A.; Huang, A.; Chen, G.; Wang, W.; Jayaseelan, J.; Katneni, K.; Patil, R.; Saunders, J.; Shahi, S. P.; Chittimalla, R.; Angulo-Barturen, I.; Jiménez-Díaz, M. B.; Wittlin, S.; Tumwebaze, P. K.; Rosenthal, P. J.; Cooper, R. A.; Aguiar, A. C. C.; Guido, R. V. C.; Pereira, D. B.; Mittal, N.; Winzeler, E. A.; Tomchick, D. R.; Laleu, B.; Burrows, J. N.; Rathod, P. K.; Fidock, D. A.; Charman, S. A.; Phillips, M. A. *J. Med. Chem.* **2021**, *64*, 6085–6136. Perry, M. W. D.; Björhall, K.; Bold, P.; Brűlls, M.; Börjesson, U.; Carlsson, J.; Chang, H.-F. A.; Chen, Y.; Eriksson, A.; Fihn, B.-M.; Fransson, R.; Fredlund, L.; Ge, H.; Huang, H.; Karabelas, K.; Lamm Bergström, E.; Lever, S.; Lindmark, H.; Mogemark, M.; Nikitidis, A.; Palmgren, A.-P.; Pemberton, N.; Petersen, J.; Rodrigo Blomqvist, M.; Smith, R. W.; Thomas, M. J.; Ullah, V.; Tyrchan, C.; Wennberg, T.; Westin Eriksson, A.; Yang, W.; Zhao, S.; Öster, L. *J. Med. Chem.* **2021**, *64*, 8053–8075. Williamson, D. S.; Smith, G. P.; Mikkelsen, G. K.; Jensen, T.; Acheson-Dossang, P.; Badolo, L.; Bedford, S. T.; Chell, V.; Chen, I.-J.; Dokurno, P.; Hentzer, M.; Newland, S.; Ray, S. C.; Shaw, T.; Surgenor, A. E.; Terry, L.; Wang, Y.; Christensen, K. V. *J. Med. Chem.* **2021**, *64*, 10312–10332. Harrison, L. A.; Atkinson, S. J.; Bassil, A.; Chung, C.; Grandi, P.; Gray, J. R. J.; Levernier, E.; Lewis, A.; Lugo, D.; Messenger, C.; Michon, A.-M.; Mitchell, D. J.; Preston, A.; Prinjha, R. K.; Rioja, I.; Seal, J. T.; Taylor, S.; Wall, I. D.; Watson, R. J.; Woolven, J. M.; Demont, E. H. *J. Med. Chem.* **2021**, *64*, 10742–10771. Heightman, T. D.; Berdini, V.; Bevan, L.; Buck, I. M.; Carr, M. G.; Courtin, A.; Coyle, J. E.; Day, J. E. H.; East, C.; Fazal, L.; Griffiths-Jones, C. M.; Howard, S.; Kucia-Tran, J.; Martins, V.; Muench, S.; Munck, J. M.; Norton, D.; O'Reilly, M.; Palmer, N.; Pathuri, P.; Peakman, T. M.; Reader, M.; Rees, D. C.; Rich, S. J.; Shah, A.; Wallis, N. G.; Walton, H.; Wilsher, N. E.; Woolford, A. J. A.; Cooke, M.; Cousin, D.; Onions, S.; Shannon, J.; Watts, J.; Murray, C. W. *J. Med. Chem.* **2021**, *64*, 12286–12303. Ryan, M. D.; Parkes, A. L.; Corbett, D.; Dickie, A. P.; Southey, M.; Andersen, O. A.; Stein, D. B.; Barbeau, O. R.; Sanzone, A.; Thommes, P.; Barker, J.; Cain, R.; Compper, C.; Dejob, M.; Dorali, A.; Etheridge, D.; Evans, S.; Faulkner, A.; Gadouleau, E.; Gorman, T.; Haase, D.; Holbrow-Wilshaw, M.; Krulle, T.; Li, X.; Lumley, C.; Mertins, B.; Napier, S.; Odedra, R.; Papadopoulos, K.; Roumpelakis, V.; Spear, K.; Trimby, E.; Williams, J.; Zahn, M.; Keefe, A. D.; Zhang, Y.; Soutter, H. T.; Centrella, P. A.; Clark, M. A.; Cuozzo, J. W.; Deng, B.; Hunt, A.; Sigel, E. A.; Troast, D. M.; Dejonge, B. L. M. *J. Med. Chem.* **2021**, *64*, 14377–14425. Du, L.; Wang, X.; Cui, G.; Xu, B. *Bioorg. Med. Chem.* **2021**, *29*, 115878. Long, M. F.; Capstick, R. A.; Spearing, P. K.; Engers, J. L.; Gregro, A. R.; Bollinger, S. R.; Chang, S.; Luscombe, V. B.; Rodriguez, A. L.; Cho, H. P.; Niswender, C. M.; Bridges, T. M.; Conn, P. J.; Lindsley, C. W.; Engers, D. W.; Temple, K. J. *Bioorg. Med. Chem. Lett.* **2021**, *53*, 128416. Feng, Y.; Park, H.; Bauer, L.; Ryu, J. C.; Yoon, S. O. K. *ACS Med. Chem. Lett.* **2021**, *12*, 24–29. Jo, U.; Senatorov, I. S.; Zimmermann, A.; Saha, L. K.; Murai, Y.; Kim, S. H.; Rajapakse, V. N.; Elloumi, F.; Takahashi, N.;

Schultz, C. W.; Thomas, A.; Zenke, F. T.; Pommier, Y. *Mol. Cancer Ther.* **2021**, *20*, 1431–1441.
Kawahata, W.; Asami, T.; Kiyoi, T.; Irie, T.; Kashimoto, S.; Furuichi, H.; Sawa, M. *J. Med. Chem.* **2021**, *64*, 14129–14141.

2022

Li, H.; Ouyang, S.; Zhang, Y.; Peng, K.; Fang, W.; Liu, Z.; Wang, C.-Y.; Zhang, X.; Wang, Y. *Eur. J. Med. Chem.* **2022**, *244*, 114858. Leśniak, R. K.; Nichols, R. J.; Schonemann, M.; Zhao, J.; Gajera, C. R.; Lam, G.; Nguyen, K. C.; Langston, J. W.; Smith, M.; Montine, T. J. *Eur. J. Med. Chem.* **2022**, *242*, 114693. Gong, Y.; Wu, F.-X.; Wang, M.-S.; Xu, H.-C.; Zhuo, L.-S.; Yang, G.-F.; Huang, W. *Eur. J. Med. Chem.* **2022**, *241*, 114654. Niu, J.; Bai, H.; Li, Z.; Gao, Y.; Zhang, Y.; Wang, X.; Yang, Y.; Xu, Y.; Geng, M.; Xie, Z.; Zhou, B. *Eur. J. Med. Chem.* **2022**, *238*, 114482. Mei, L.-C.; Zhuo, L.-S.; Xu, H.-C.; Huang, W.; Hao, G.-F.; Yang, G.-F. *Eur. J. Med. Chem.* **2022**, *237*, 114406. Grychowska, K.; Olejarz-Maciej, A.; Blicharz, K.; Pietruś, W.; Karcz, T.; Kurczab, R.; Koczurkiewicz, P.; Doroz-Płonka, A.; Latacz, G.; Keeri, A. R.; Piska, K.; Satała, G.; Pęgiel, J.; Trybała, W.; Jastrzębska-Więsek, M.; Bojarski, A. J.; Lamaty, F.; Partyka, A.; Walczak, M.; Krawczyk, M.; Malikowska-Racia, N.; Popik, P.; Zajdel, P. *Eur. J. Med. Chem.* **2022**, *236*, 114329. Chen, L.; Su, M.; Wu, X.-Z.; Wang, D.-Z.; Kang, Y.; Wang, C.-G.; Assani, I.; Wang, M.-X.; Zhao, S.-F.; Lv, S.-M.; Wang, J.-W.; Sun, B.; Li, Y.; Jin, Q.; Huang, R.-Z.; Liao, Z.-X. *Eur. J. Med. Chem.* **2022**, *229*, 114065. Yan, Y.-H.; Li, W.; Chen, W.; Li, C.; Zhu, K.-R.; Deng, J.; Dai, Q.-Q.; Yang, L.-L.; Wang, Z.; Li, G.-B. *Eur. J. Med. Chem.* **2022**, *228*, 113965. Wei, W.; Feng, Z.; Liu, Z.; Li, X.; He, H.; Ran, K.; Shi, Y.; Zhu, Y.; Ye, T.; Gao, C.; Wang, N.; Yu, L. *Eur. J. Med. Chem.* **2022**, *228*, 113978. Lu, Y.; Feng, Y.; Li, Z.; Li, J.; Zhang, H.; Hu, X.; Jiang, W.; Shi, T.; Wang, Z. *Eur. J. Med. Chem.* **2022**, *227*, 113908. Griffith, D. A.; Edmonds, D. J.; Fortin, J.-P.; Kalgutkar, A. S.; Kuzmiski, J. B.; Loria, P. M.; Saxena, A. R.; Bagley, S. W.; Buckeridge, C.; Curto, J. M.; Derksen, D. R.; Dias, J. M.; Griffor, M. C.; Han, S.; Jackson, V. M.; Landis, M. S.; Lettiere, D.; Limberakis, C.; Liu, Y.; Mathiowetz, A. M.; Patel, J. C.; Piotrowski, D. W.; Price, D. A.; Ruggeri, R. B.; Tess, D. A. *J. Med. Chem.* **2022**, *65*, 8208–8226. Shearer, J.; Castro, J. L.; Lawson, A. D. G.; MacCoss, M.; Taylor, R. D. *J. Med. Chem.* **2022**, *65*, 8699–8712. Shinde, A.; Ugale, S. R.; Nandurkar, Y.; Modak, M.; Chavan, A. P.; Mhaske, P. C. *ACS Omega* **2022**, *7*, 47096–47107. Hopkins, B. T.; Bame, E.; Bajrami, B.; Black, C.; Bohnert, T.; Boiselle, C.; Burdette, D.; Burns, J. C.; Delva, L.; Donaldson, D.; Grater, R.; Gu, C.; Hoemberger, M.; Johnson, J.; Kapadnis, S.; King, K.; Lulla, M.; Ma, B.; Marx, I.; Magee, T.; Meissner, R.; Metrick, C. M.; Mingueneau, M.; Murugan, P.; Otipoby, K. L.; Polack, E.; Poreci, U.; Prince, R.; Roach, A. M.; Rowbottom, C.; Santoro, J. C.; Schroeder, P.; Tang, H.; Tien, E.; Zhang, F.; Lyssikatos, J. *J. Med. Chem.* **2022**, *65*, 1206–1224. Keylor, M. H.; Gulati, A.; Kattar, S. D.; Johnson, R. E.; Chau, R. W.; Margrey, K. A.; Ardolino, M. J.; Zarate, C.; Poremba, K. E.; Simov, V.; Morriello, G. J.; Acton, J. J.; Pio, B.; Yan, X.; Palte, R. L.; McMinn, S. E.; Nogle, L.; Lesburg, C. A.; Adpressa, D.; Lin, S.; Neelamkavil, S.; Liu, P.; Su, J.; Hegde, L. G.; Woodhouse, J. D.; Faltus, R.; Xiong, T.; Ciaccio, P. J.; Piesvaux, J.; Otte, K. M.; Wood, H. B.; Kennedy, M. E.; Bennett, D. J.; DiMauro, E. F.; Fell, M. J.; Fuller, P. H. *J. Med. Chem.* **2022**, *65*, 838–856. Casimiro-Garcia, A.; Allais, C.; Brennan, A.; Choi, C.; Dower, G.; Farley, K. A.; Fleming, M.; Flick, A.; Frisbie, R. K.; Hall, J.; Hepworth, D.; Jones, H.; Knafels, J. D.; Kortum, S.; Lovering, F. E.; Mathias, J. P.; Mohan, S.; Morgan, P. M.; Parng, C.; Parris, K.; Pullen, N.; Schlerman, F.; Stansfield, J.; Strohbach, J. W.; Vajdos, F. F.; Vincent, F.; Wang, H.; Wang, X.; Webster, R.; Wright, S. W. *J. Med. Chem.* **2022**, *65*, 757–784. Jecs, E.; Tahirovic, Y. A.; Wilson, R. J.; Miller, E. J.; Kim, M.; Truax, V.; Nguyen, H. H.; Akins, N. S.; Saindane, M.; Wang, T.; Sum, C. S.; Cvijic, M. E.; Schroeder, G. M.; Burton, S. L.; Derdeyn, C. A.; Xu, L.; Jiang, Y.; Wilson, L. J.; Liotta, D. C. *J. Med. Chem.* **2022**, *65*, 4058–4084. Frankowski, K. J.; Patnaik, S.; Wang, C.; Southall, N.; Dutta, D.; De, S.; Li, D.; Dextras, C.; Lin, Y.-H.; Bryant-Connah, M.; Davis, D.; Wang, F.; Wachsmuth, L. M.; Shah, P.; Williams, J.; Kabir, M.; Zhu, E.; Baljinnyam, B.; Wang, A.; Xu, X.; Norton, J.; Ferrer, M.; Titus, S.; Simeonov, A.; Zheng, W.; Mathews Griner, L. A.; Jadhav, A.; Aubé, J.; Henderson, M. J.; Rudloff, U.; Schoenen, F. J.; Huang, S.; Marugan, J. J. *J. Med. Chem.* **2022**, *65*, 8303–8331. Hartz, R. A.; Xu, L.; Sit, S.-Y.; Chen, J.; Venables, B. L.; Lin, Z.; Zhang, S.; Li, Z.; Parker, D.; Simmons, T. S.; Jenkins, S.; Hanumegowda, U. M.; Dicker, I.; Krystal, M.; Meanwell, N. A.; Regueiro-Ren, A. *J. Med. Chem.* **2022**, *65*, 15935–15966. Meibom, D.; Micus, S.; Andreevski, A. L.; Anlauf, S.; Bogner, P.; von Buehler, C.-J.; Dieskau, A. P.; Dreher, J.; Eitner, F.; Fliegner, D.; Follmann, M.; Gericke, K. M.; Maassen, S.; Meyer, J.; Schlemmer, K.-H.; Steuber, H.; Tersteegen, A.; Wunder, F. *J. Med. Chem.* **2022**, *65*, 16420–16431. Goldberg, F. W.; Ting, A. K. T.; Beattie, D.; Lamont, G. M.; Fallan, C.; Finlay, M. R. V.; Williamson, B.; Schimpl, M.; Harmer, A. R.; Adeyemi, O. B.; Nordell, P.; Cronin, A. S.; Vazquez-Chantada, M.; Barratt, D.; Ramos-Montoya, A.; Cadogan, E. B.; Davies, B. R. *ACS Med. Chem. Lett.* **2022**, *13*, 1295–1301. Xue, D.; Xu, Y.; Kyani, A.; Roy, J.; Dai, L.; Sun, D.; Neamati, N. *J. Med. Chem.* **2022**, *65*, 343–368. Mata, G.; Miles, D. H.; Drew, S. L.; Fournier, J.; Lawson, K. V.; Mailyan, A. K.; Sharif, E. U.; Yan, X.; Beatty, J. W.; Banuelos,

J.; Chen, J.; Ginn, E.; Chen, A.; Gerrick, K. Y.; Pham, A. T.; Wong, K.; Soni, D.; Dhanota, P.; Shaqfeh, S. G.; Meleza, C.; Narasappa, N.; Singh, H.; Zhao, X.; Jin, L.; Schindler, U.; Walters, M. J.; Young, S. W.; Walker, N. P.; Leleti, M. R.; Powers, J. P.; Jeffrey, J. L. *J. Med. Chem.* **2022**, *65*, 1418–1444. Xue, D.; Xu, Y.; Kyani, A.; Roy, J.; Dai, L.; Sun, D.; Neamati, N. *J. Med. Chem.* **2022**, *65*, 3404–3419. Li, X.; Zhang, Z.; Chen, Y.; Wang, B.; Yang, G.; Xu, X.; Yechao, B.; Bai, D.; Feng, B.; Mao, Y.; Feng, J.; Bai, C.; He, F.; Tao, W. *ACS Med. Chem. Lett.* **2022**, *13*, 507–512. Verma, V. A.; Wang, L.; Labadie, S. S.; Liang, J.; Sellers, B. D.; Wang, J.; Dong, L.; Wang, Q.; Zhang, S.; Xu, Z.; Zhang, Y.; Niu, Y.; Wang, X.; Wai, J.; Koehler, M. F. T.; Hu, H.; Alexander, M. K.; Nishiyama, M.; Miu, A.; Xu, Y.; Pang, J.; Katakam, A. K.; Reichelt, M.; Austin, C. D.; Ho, H.; Payandeh, J.; Koth, C. M. *J. Med. Chem.* **2022**, *65*, 4085–4120. Tamayo, N. A.; Bourbeau, M. P.; Allen, J. R.; Ashton, K. S.; Chen, J. J.; Kaller, M. R.; Nguyen, T. T.; Nishimura, N.; Pettus, L. H.; Walton, M.; Belmontes, B.; Moriguchi, J.; Chen, K.; McCarter, J. D.; Hanestad, K.; Chung, G.; Ninniri, M. S. S.; Sun, J.; Poppe, L.; Spahr, C.; Hui, J.; Jia, L.; Wu, T.; Dahal, U. P.; Edson, K. Z.; Payton, M. *J. Med. Chem.* **2022**, *65*, 4972–4990. Yin, W.; Wu, T.; Liu, L.; Jiang, H.; Zhang, Y.; Cui, H.; Sun, Y.; Qin, Q.; Sun, Y.; Gao, Z.; Zhao, L.; Su, X.; Zhao, D.; Cheng, M. *J. Med. Chem.* **2022**, *65*, 5539–5564. Chen, P.; Bin, H.; Jiao, Y.; Lin, G.; Zhang, Y.; Xia, A.; Pan, Z.; Qiao, J.; Guo, Y.; Liu, J.; Zhou, Y.; Li, L. *Bioorg. Med. Chem. Lett.* **2022**, *63*, 128651. Caroff, E.; Meyer, E. A.; Äänismaa, P.; Froidevaux, S.; Keller, M.; Piali, L. *J. Med. Chem.* **2022**, *65*, 11533–11549. Gao, Y.; Wang, H.; Shen, L.; Xu, H.; Deng, M.; Cheng, M.; Wang, J. *Bioorg. Chem.* **2022**, *123*, 105769. Chen, D.; Tan, X.; Chen, W.; Liu, Y.; Li, C.; Wu, J.; Zheng, J.; Shen, H. C.; Zhang, M.; Wu, W.; Wang, L.; Xiong, J.; Dai, J.; Sun, K.; Zhang, J. D.; Xiang, K.; Li, B.; Ni, X.; Zhu, Q.; Gao, L.; Wang, L.; Feng, S. *J. Med. Chem.* **2022**, *65*, 10938–10955. Pandit, N.; Yoo, M.; Hyun Park, T.; Kim, J.; Mi Kim, S.; Myung Lee, K.; Kim, Y.; Min Bong, S.; Il Lee, B.; Jung, K.-Y.; Hoon Park, C. *Bioorg. Med. Chem.* **2022**, *72*, 116967. Pujala, B.; Ramachandran, S. A.; Sonawane, M.; Kamble, M. M.; Panpatil, D.; Adhikari, S.; Soni, S.; Subbareddy, V.; Shinde, B. U.; Nayak, A. K.; Bansal, C.; Gupta, A.; Mukherjee, K.; Agarwal, A. K.; Guerrero, J.; Herrera, F. J.; Bernales, S.; Guha, M.; Chakravarty, S.; Pham, S. M.; Rai, R. *Bioorg. Med. Chem. Lett.* **2022**, *75*, 128979. Shvartsbart, A.; Roach, J. J.; Witten, M. R.; Koblish, H.; Harris, J. J.; Covington, M.; Hess, R.; Lin, L.; Frascella, M.; Truong, L.; Leffet, L.; Conlen, P.; Beshad, E.; Klabe, R.; Katiyar, K.; Kaldon, L.; Young-Sciame, R.; He, X.; Petusky, S.; Chen, K.-J.; Horsey, A.; Lei, H.-T.; Epling, L. B.; Deller, M. C.; Vechorkin, O.; Yao, W. *J. Med. Chem.* **2022**, *65*, 15433–15442. Li, D.; Sloman, D. L.; Achab, A.; Zhou, H.; McGowan, M. A.; White, C.; Gibeau, C.; Zhang, H.; Pu, Q.; Bharathan, I.; Hopkins, B.; Liu, K.; Ferguson, H.; Fradera, X.; Lesburg, C. A.; Martinot, T. A.; Qi, J.; Song, Z. J.; Yin, J.; Zhang, H.; Song, L.; Wan, B.; DAddio, S.; Solban, N.; Miller, J. R.; Zamlynny, B.; Bass, A.; Freeland, E.; Ykoruk, B.; Hilliard, C.; Ferraro, J.; Zhai, J.; Knemeyer, I.; Otte, K. M.; Vincent, S.; Sciammetta, N.; Pasternak, A.; Bennett, D. J.; Han, Y. *J. Med. Chem.* **2022**, *65*, 6001–6016. Fuerst, R.; Choi, J. Y.; Knapinska, A. M.; Cameron, M. D.; Ruiz, C.; Delmas, A.; Sundrud, M. S.; Fields, G. B.; Roush, W. R. *Bioorg. Med. Chem. Lett.* **2022**, *76*, 129014. Huff, S.; Kummetha, I. R.; Zhang, L.; Wang, L.; Bray, W.; Yin, J.; Kelley, V.; Wang, Y.; Rana, T. M. *J. Med. Chem.* **2022**, *65*, 10920–10937. Wilson, C.; Ray, P.; Zuccotto, F.; Hernandez, J.; Aggarwal, A.; Mackenzie, C.; Caldwell, N.; Taylor, M.; Huggett, M.; Mathieson, M.; Murugesan, D.; Smith, A.; Davis, S.; Cocco, M.; Parai, M. K.; Acharya, A.; Tamaki, F.; Scullion, P.; Epemolu, O.; Riley, J.; Stojanovski, L.; Lopez-Román, E. M.; Torres-Gómez, P. A.; Toledo, A. M.; Guijarro-Lopez, L.; Camino, I.; Engelhart, C. A.; Schnappinger, D.; Massoudi, L. M.; Lenaerts, A.; Robertson, G. T.; Walpole, C.; Matthews, D.; Floyd, D.; Sacchettini, J. C.; Read, K. D.; Encinas, L.; Bates, R. H.; Green, S. R.; Wyatt, P. G. *J. Med. Chem.* **2022**, *65*, 409–423. Owens, T. D.; Brameld, K. A.; Verner, E. J.; Ton, T.; Li, X.; Zhu, J.; Masjedizadeh, M. R.; Bradshaw, J. M.; Hill, R. J.; Tam, D.; Bisconte, A.; Kim, E. O.; Francesco, M.; Xing, Y.; Shu, J.; Karr, D.; LaStant, J.; Finkle, D.; Loewenstein, N.; Haberstock-Debic, H.; Taylor, M. J.; Nunn, P.; Langrish, C. L.; Goldstein, D. M. *J. Med. Chem.* **2022**, *65*, 5300–5316.
